# Explosive Tandem and Segmental Duplications of Multigenic Families in *Eucalyptus grandis*

**DOI:** 10.1093/gbe/evv048

**Published:** 2015-03-13

**Authors:** Qiang Li, Hong Yu, Phi Bang Cao, Nizar Fawal, Catherine Mathé, Sahar Azar, Hua Cassan-Wang, Alexander A. Myburg, Jacqueline Grima-Pettenati, Christiane Marque, Chantal Teulières, Christophe Dunand

**Affiliations:** ^1^Laboratoire de Recherche en Sciences Végétales, UPS, UMR 5546, Université de Toulouse, Castanet-Tolosan, France; ^2^CNRS, UMR 5546, Castanet-Tolosan, France; ^3^Department of Genetics, Forestry and Agricultural Biotechnology Institute (FABI), University of Pretoria, South Africa; ^4^Genomics Research Institute (GRI), University of Pretoria, South Africa

**Keywords:** multigenic families, gene duplication, phylogenetic analysis, gene structures, chromosomal localization, gene annotation

## Abstract

Plant organisms contain a large number of genes belonging to numerous multigenic families whose evolution size reflects some functional constraints. Sequences from eight multigenic families, involved in biotic and abiotic responses, have been analyzed in *Eucalyptus grandis* and compared with *Arabidopsis thaliana.* Two transcription factor families APETALA 2 (AP2)/ethylene responsive factor and GRAS, two auxin transporter families PIN-FORMED and AUX/LAX, two oxidoreductase families (ascorbate peroxidases [APx] and Class III peroxidases [CIII Prx]), and two families of protective molecules late embryogenesis abundant (LEA) and DNAj were annotated in expert and exhaustive manner. Many recent tandem duplications leading to the emergence of species-specific gene clusters and the explosion of the gene numbers have been observed for the AP2, GRAS, LEA, PIN, and CIII Prx in *E. grandis*, while the APx, the AUX/LAX and DNAj are conserved between species. Although no direct evidence has yet demonstrated the roles of these recent duplicated genes observed in *E. grandis,* this could indicate their putative implications in the morphological and physiological characteristics of *E. grandis,* and be the key factor for the survival of this nondormant species. Global analysis of key families would be a good criterion to evaluate the capabilities of some organisms to adapt to environmental variations.

## Introduction

In plants, 30% of the genes are multigenic family members. Among these families, some have undergone intensive expansions, others were submitted to a strong selection pressure to maintain them with similar numbers, with a very low divergence rate, across different plant genomes (Armisen et al. 2008). Plant lifestyle, environmental adaptations and numerous duplication or transposition events can explain the large multigenic families found in plants (Freeling 2009). Duplicated genes are not always conserved and can become pseudogenes. The global analysis conducted on paralogous pairs of regulatory genes in *Arabidopsis thaliana* showed that in a large majority of cases, expression significantly differs within organs between paralogs which is in favor of subfunctionalization and neofunctionalization after duplications (Duarte et al. 2006).

In the same way, a striking result of comparative genomics showed that gene birth and death occur with rates similar to those of nucleotide substitutions per site (Taylor and Raes 2004; Demuth and Hahn 2009). This suggests that duplication plays an important role in the adaptation process, as well as the sequence divergence between orthologs. An arising question concerns the chronology of events: Are duplication events the result of a large adaptation process? Or did the many duplication events allow changes in plant lifestyle? Most likely, the current situation is the result of a “zig-zag dialog” between genome plasticity and environmental adaptation.

In order to bring answers, several large multigenic families of *Eucalyptus grandis*, the most widely planted tree species characterized by a fast-growing development and recently sequenced (Myburg et al. 2014), have been annotated. It allows the genomic comparison with the *A*. *thaliana* and *Populus trichocarpa*. Eight multigenic families of various sizes have been analyzed in order to obtain a gene list as accurate and complete as possible and to correlate duplication events and species evolution.

### APETALA 2/Ethylene Responsive Factor Family

The AP2/ERF (APETALA 2/ethylene responsive factor) is a large family of plant-specific transcription factors involved in developmental regulations and responses to biotic and abiotic stresses. Based on the number of AP2 binding domains, the AP2/ERF family is divided into five classes (Sakuma et al. 2002): AP2, RAV (related to ABI3/VP1), ERF, DREB (dehydration responsive element binding), and a soloist. The AP2 proteins are reported to be involved in the regulation of plant development whereas the RAV proteins participate in biotic and abiotic stress responses. The ERF subfamily constitutes the largest group of genes found to be involved in abiotic stress responses through ethylene-dependent or -independent pathways. However, the functions of abiotic stress-inducible ERF genes are still unknown. In contrast, it is admitted that the DREB genes are major factors in plant abiotic stress responses by activating the expression of many genes via the dehydration-responsive-element/c-repeat cis-element (Lata and Prasad 2011).

### Auxin Transporters: PIN and AUX/LAX Families

The hormone auxin plays a crucial role in control of plant growth/development and response to environmental stimuli. As the auxin response in plant is highly dependent on auxin transport, its disruption impacts the majority of auxin-related developmental processes. Two types of auxin transporters were identified: auxin influx carrier AUX1/LAX (like AUX1) family and efflux carrier PIN-FORMED (PIN) family. Our knowledge of auxin transport in plant development is mainly obtained from the model plant *A. thaliana* and some other herbaceous plants such as maize, but little from woody plants, particularly concerning the role of auxin transport in wood formation, a developmental process specific to woody plants. Indeed, it has been well demonstrated that there is a high level of auxin in cambium that decreases almost to zero in the mature xylem or phloem cells in poplar and pinus (Uggla et al. 1996; Tuominen et al. 1997).

### DNAj/HSP40 Family

DNAj proteins, also called HSP40 (heat shock protein 40 kDa), form a large and diverse protein family expressed in most of the organisms including plants (Qiu et al. 2006). They contain an N-term highly conserved domain of 70-amino acids (J-domain) and a low similarity region of 120–170 residues at the C-terminal (Bork et al. 1992). Based on their structure, the DNAj proteins are classified into four types (Cheetham and Caplan 1998). In the plant kingdom, they diversely function in developmental processes and stress responses, such as folding, unfolding, protein transport, and degradation by interacting with HSP70, another molecular chaperone, and by stimulating its ATPase activity (Wang et al. 2004; Yang et al. 2010).

### GRAS Family

GRAS is a family of plant-specific transcriptional factors, containing eight subfamilies: DELLA, HAIRY MERISTEM (HAM), LiSCL (L. longiflorum SCARECROW-like)PHYTOCHROME A SIGNAL TRANSDUCTION1 (PAT1), LATERAL SUPPRESSOR (LS), SCARECROW (SCR), SHORTROOT (SHR), SCARECROW-LIKE 3 (SCL3) (Bolle 2004). The GRAS proteins show conserved residues in C-terminal and a variable N-terminal domain (Hirsch and Oldroyd 2009). GRAS may have a role in plant development, shoot apical meristem maintenance (Bolle 2004; Lee et al. 2008) and participate in the plant response to abiotic stresses and nodulation signaling in *Medicago truncatula* (Liu et al. 2011).

### Late Embryogenesis Abundant Family

The late embryogenesis abundant (LEA) proteins, initially found in plants, are also detected in other kingdoms (Reardon et al. 2010; Su et al. 2011). LEAs are classified into eight subfamilies: LEA1 to 6, dehydrin and Seed Maturation Protein (SMP). This family underwent rapid expansion during the early evolution of land plants. In plants, the LEAs accumulate during late embryogenesis and in vegetative tissues exposed to dehydration, cold, salt, or abscisic acid treatment (Yakovlev et al. 2008).

Highly hydrophilic and amphiphilic, the LEAs can prevent the aggregation of proteins, and the irreversible denaturation of membranes and proteins which can be observed during drought or salt stress (Kosová et al. 2011; Olvera-Carrillo et al. 2011).

### Peroxidase Families: Ascorbate Peroxidases and Class III Peroxidases

Ascorbate peroxidases (APx) and Class III peroxidases (CIII Prx) families belong to the nonanimal peroxidase superfamily and catalyze red-ox reactions (Passardi et al. 2004). APx are detected in all chloroplast containing organisms and play a key role in H_2_O_2_ homeostasis (Mano and Asada 1999). They form a small multigenic family well conserved within divergent organisms which will be a good control for interspecies duplication events. CIII Prxs form a large multigenic family in higher plants and participate in many different processes such as auxin metabolism, cell wall elongation, stiffening, and protection against pathogens (Passardi et al. 2004).

Among the 36,376 genes identified in the *E. grandis* genome, this article presents the expert annotation of more than 700 genes from eight multigenic families. The comparison with the same families from *A. thaliana*, *P. trichocarpa,* and *Vitis vinifera* allowed the analysis of duplication events in the process of evolution. Finally, through genome localization and phylogenetic analysis between members of *E. grandis* and *A. thaliana*, we studied tandem, segmental and whole-genome duplication (WGD) events of these gene families in *E. grandis*.

## Materials and Methods

### Sources of Genomic and Protein Sequences

The *E. grandis* genome and proteome, available at Phytozome, (http://www.phytozome.net/eucalyptus.php, last accessed March 25, 2015) were downloaded using the first version of the JGI. Peroxidase sequences from *A. thaliana* or *P. trichocarpa* are available at the PeroxiBase (http://peroxibase.toulouse.inra.fr, last accessed March 25, 2015). The *P. trichocarpa* AP2/ERF family gene annotations were taken from Zhuang et al. (2008) and from Licausi et al. (2010) for *V. vinifera*.

### Datamining and Annotation

Exhaustive and expert annotation was performed as following to discard prediction errors inherent to automatic annotations (Fawal et al. 2014). First, BLASTP was performed between the whole *E. grandis* proteome and the already annotated sequences from *P. trichocarpa.* The obtained protein batches corresponding to the different protein families were manually analyzed based on the presence of the characteristic domain of each family. Alternative transcript variants and redundant sequences were discarded to prevent artifacts during phylogenetic analysis. Partial gene models were verified based on gene structures, presences of conserved domains and EST (expressed sequence tag) supports. These corrected sets of proteins were used to determine the corresponding chromosomal positions, gene structures, and coding sequences using the spliced alignment program Scipio (Keller et al. 2008). New paralogous sequences, initially not annotated, were found thanks to this method and integrated in the final batch of proteins. Each gene has been numbered as following: *Egr*, followed by the protein abbreviation and by a number representing the order of the position on the chromosomes.

Regarding the gene families from *A. thaliana*, *P. trichocarpa*, and *V. vinifera*, data were obtained from literature when available. For the DNAj family, since no exhaustive data were available, the annotation has been done for the four organisms.

### Phylogenetic and Clustering Analysis

All protein sequences can be found in the PeroxiBase (http://peroxibase.toulouse.inra.fr; Fawal et al. 2013) and in EucaToul (http://www.polebio.lrsv.ups-tlse.fr/eucatoul/index.php). Complete sequences were aligned using MAFFT (Thompson et al. 1994) and further inspected and visually adjusted using BioEdit 7.2 (Tippmann 2004). The phylogenetic trees were reconstructed with the maximum-likelihood (ML) method using PhyML version 3.0 (Guindon et al. 2010). The substitution model determined by protTest (Abascal et al. 2005) was LG (Le and Gascuel 2008) and a gamma distribution (four discrete categories of sites and an estimated alpha parameter) was used. The ML algorithm BIONJ (Gascuel 1997) distance-based tree was used to refine the starting tree. The latter tree was optimized for topology, branch lengths, and substitution rate parameters using the approximate likelihood ratio test (aLRT). The aLRT statistics assess the likelihood that a branch exists on a ML tree (Anisimova and Gascuel 2006). The nonparametric Shimodaira–Hasegawa-like procedure was used to interpret the aLRT statistics by converting them to bootstrap values. Trees were edited and analyzed using TreeDyn (Chevenet et al. 2006) and Archaeopteryx (Han and Zmasek 2009). Finally, species-specific clusters were collapsed to facilitate the tree interpretations.

### Genomic Comparison

The intron/exon coordinates together with the corresponding genomic sequences of all identified genes were determined with Scipio (Keller et al. 2008). The intron/exon organization of the different families was verified with CIWOG (Wilkerson et al. 2009), and GECA (Fawal et al. 2012) to support the correct annotation.

Graphical presentation of gene localization on chromosomes and linkage between genes were produced using MapChart V2.1 (Voorrips 2002).

### Duplication Events and Expression Analysis

Gene family expansion is associated with WGDs, segmental duplications (SDs), and tandem duplications (TDs). Different definitions are available for these events, and in order to analyze them, we have defined them as following: WGD as blocks of DNA that map to different loci in another chromosome, SD as blocks of DNA that map to different loci in the same chromosome and TD as clusters of duplicated genes. Duplication events were highlighted thanks to the combined phylogenetic analysis of *A. thaliana* and *E. grandis*. The analysis of the orthologous and paralogous relationships has allowed determining the existing duplications. Based on the definitions made above, the distinction between WGD, SD, and TD has been made thanks to the analysis of chromosomal localization.

To analyze the relationship between gene duplication and gene functionalization, the RNA-seq data were visualized and analyzed using Expander version 6 (Ulitsky et al. 2010). *Eucalyptus grandis* EST library available from NCBI were also analyzed.

## Results

**Fig. 1.—:**
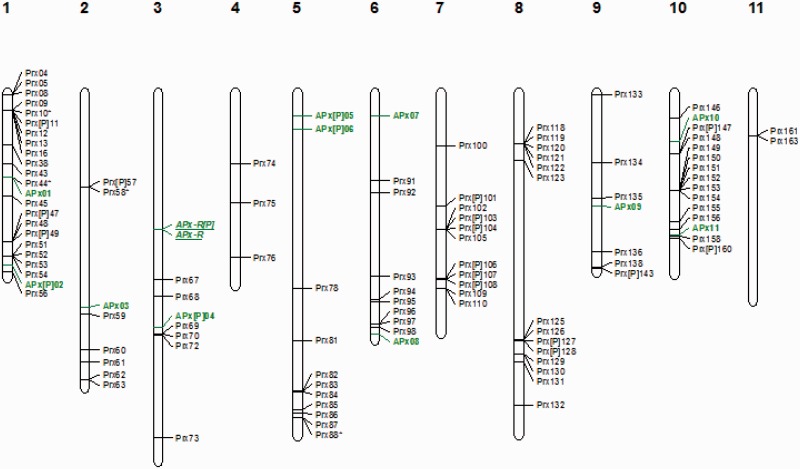
Genomic localization of Peroxidase gene family from *E. grandis* without reannotation. APx and CIII Prx genes obtained from an automatic annotation including complete sequences, partial sequences, and pseudogenes are presented. APx genes are marked in green including APx-R which are in italic and underlined. CIII Prx genes are marked in black.

Thanks to this family focused analysis, over 700 genes have been annotated in *E. grandis* genome, meaning that 2% of the genome has been expertly annotated during this work (supplementary tables S1–S7, Supplementary Material online) and compared with *A. thaliana*, *P. trichocarpa,* and *V. vinifera* (table 1). The manual and deep annotations allowed pinpointing of the weaknesses of automatic annotations. Indeed, all analyzed families have required reannotation work ranging from 19% to 67% of the gene family (table 2). This reannotation work is considered to be light if only a short 5′-end is missing or heavy when a large part of the protein sequence or a whole sequence are missing due to an incorrect prediction. The impact of the reannotated duplicated genes is major regarding the intra- and interspecies evolution analysis (tables 1 and 2 and fig. 1).

**Table 1:** AP2, GRAS, PIN, AUX/LAX, CIII Prx, and APx Isoform Numbers Found in Four Dicotyledonous Organisms

Organisms	Genes	AP2/ ERF	PIN	AUX/LAX	DNAj/ HS40	GRAS	LEA	APx	CIII Prx	Thiol Prx	Kat
*Eucalyptus* ***grandis***	36,376	202 (11)	17 (2)	5	101 (2)	92 (3)	129 (3)	13 (5)	181 (47)	17	12 (5)
*Arabidopsis* ***thaliana***	21,189	147	8	4	115	33	93	9 (1)	75 (2)	18 (1)	3
*Populus* ***trichocarpa***	30,260	200	16	8	140	98	93	11 (1)	99 (12)	18 (4)	4 (1)
*Vitis* ***vinifera***	21,189	149	9	4	88	46	42	10 (2)	97 (10)	13 (1)	2

Note.—The data from *E. grandis* were obtained from predicted proteome and the manual annotations of the predictions. When not found in the literature, data from *A. thaliana*, *P. trichocarpa* and *V. vinifera* were obtained as for *E. grandis*. Theoretical translation or pseudogene (sequence with missing motifs, with stop codon in frame and with gap in the sequence) which had been counted in the total are notified in brackets.

**Table 2:** Automatic versus Manual Annotation

Family	Annotated by Phytozome^a^	Annotated Manually^b^	Total No^c^	Ratio of Reannotation (%)
**AP2/ERF**	189 (84)	97	202 (11)	48
**GRAS**	92 (18)	18	92 (3)	20
**PIN**	15 (3)	5	17 (2)	29
**AUX/LAX**	5 (1)	1	5	20
**ARF**	17 (5)	5	17	29
**IAA**	24 (5)	5	26	19
**Apx**	13 (6)	6	13 (5)	46
**CIII Prx**	94 (31)	118	181 (47)	65
**LEA**	111 (29)	47	129 (3)	36
**DNAj/HSP40**	97 (14)	18	101 (2)	18

^a^Including correctly and incorrectly annotated sequences. The number of incorrect annotations is noted in brackets.

^b^The number of manually annotated sequences due to bad and partial prediction, lack of prediction, or withdrawal of accession between two successive Phytozome versions.

^c^Theoretical translation or pseudogene is noted in the bracket. As some genes annotated as pseudogenes contain undetermined residues, they may turn into true genes with a future sequence release.

### APETALA 2/Ethylene Responsive Factor

**Fig. 2.—:**
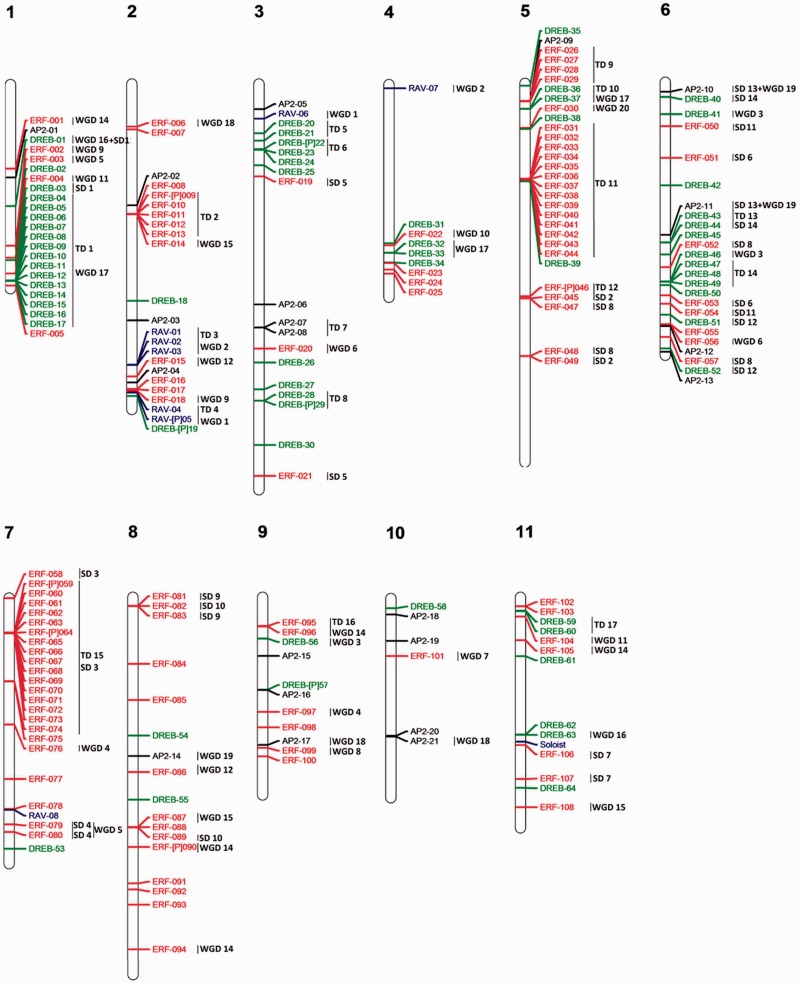
Genomic localization of AP2/ERF genes from *E. grandis*. All the predicted AP2 genes including complete sequences, partial sequences and pseudogenes are presented. ERF genes are marked in red, AP2 in black, DREB in green, and RAV and soloist in blue. TD clusters, SD events, and WGD events are displayed on the right side of the corresponding sequences or segments.

Two hundred and two AP2 sequences can be detected in the *E. grandis* genome and half of them have required a reannotation. The gene number is similar to *P. trichocarpa* but significantly larger than in *A. thaliana* and *V. vinifera* (Sakuma et al. 2002; Feng et al. 2005; Zhuang et al. 2008; Licausi et al. 2010) mainly due to recent TDs and older SDs of the ERF and DREB subfamilies (table 1 and supplementary fig. S1, Supplementary Material online). AP2/ERF genes are unevenly distributed on the 11 chromosomes of *E. grandis* and are present in all regions of the chromosomes. Hot spots of AP2/ERF duplication events (mix of recent TDs and older SDs) are mainly located in a small region of the chromosome 1 (14 DREB), 5 and 7 (22 and 19 ERF, respectively; fig. 2 and supplementary fig. S1, Supplementary Material online).

### Auxin Transporters: PIN and AUX/LAX

**Fig. 3.—:**
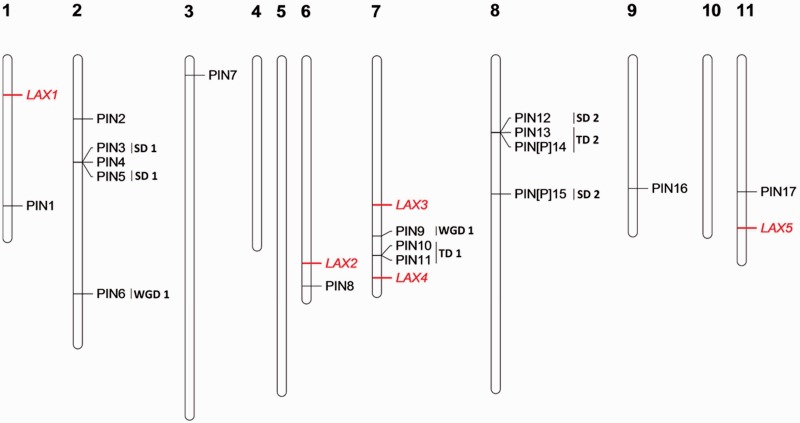
Genomic localization of auxin transporters PIN and AUX/LAX genes from *E. grandis*. All the predicted PIN (black) and AUX/LAX (red) genes are presented. TD clusters, SD events, and WGD events are displayed on the right side of the corresponding sequences or segments.

Seventeen complete PIN genes and 5 AUX/LAX genes were detected in *E. grandis* and 27% required a reannotation (supplementary table S2, Supplementary Material online). The size of the PIN family in *E. grandis* is similar to *P. trichocarpa* and much larger compared with *A. thaliana* and *V. vinifera* mainly due to an extension of short PIN from group II. The small AUX/LAX family remains similar in the isoform numbers in *E. grandis* (5), *A. thaliana* (4), and *V. vinifera* (4), whereas it almost doubles in *P. trichocarpa* (8) (table 1 and supplementary fig. S3, Supplementary Material online). In silico mapping of these genes’ loci shows that *EgrPIN* genes are located on 8 of the 11 chromosomes. Two TDs, two SDs, and one WGDs containing in total ten genes were identified (fig. 3). However, five *EgrAUX* were mapped on 4 of the 11 chromosomes without any TDs. Interestingly, the “short” PINs have been shown to be predominantly targeted to the endoplasmic reticulum, where they regulate subcellular auxin compartmentalization and homeostasis.

### DNAj/HSP40 family

**Fig. 4.—:**
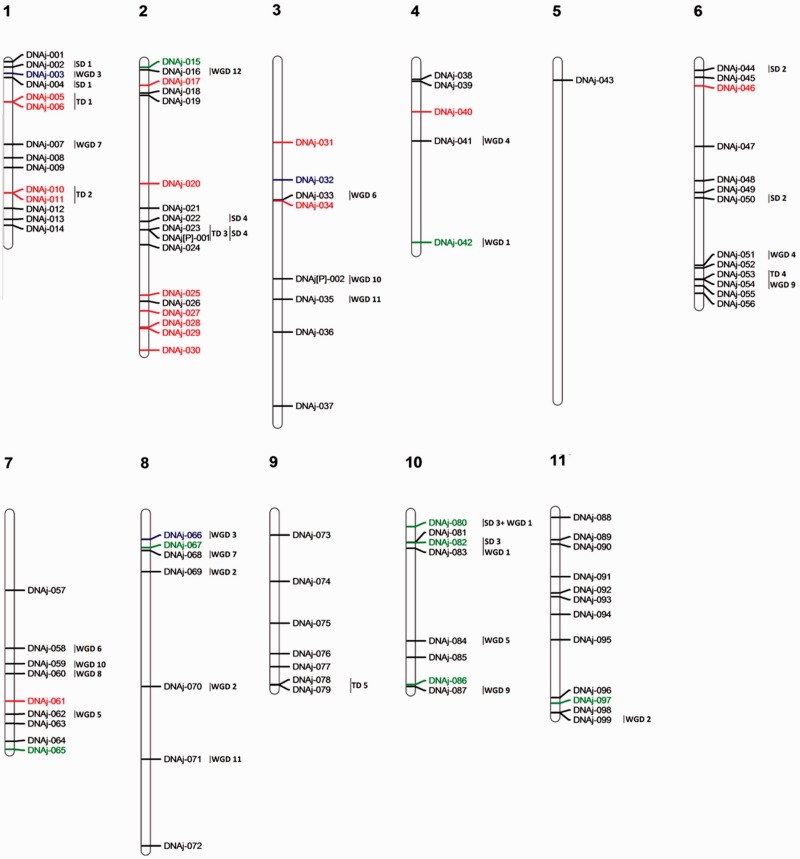
Genomic localization of DNAj genes from *E. grandis*. All the predicted DNAj genes including complete sequences, partial sequences, and pseudogenes are presented. DNAj Type I genes are marked in green, DNAj Type II genes in red, DNAj Type III genes in black, and DNAj IV JLP2 genes in blue. TD clusters, SD events, and WGD events are displayed on the right side of the corresponding sequences or segments.

Due to the lack of global analysis, a particular annotation effort has been required for the annotation of DNAj in the four organisms (table 1). One hundred and one DNAj isoforms have been detected in *E. grandis* belonging to four types and 18% required a reannotation (supplementary table S3, Supplementary Material online). The DNAj family is conserved between the four species. In *E. grandis*, DNAj genes are unevenly distributed on the genome (fig. 4 and supplementary fig. S4, Supplementary Material online). The explosion of Type III could be due to old duplications because most of the time *E. grandis* orthologs can be found in *A. thaliana* (supplementary fig. S4, Supplementary Material online) and no duplication hot spots are observed (fig. 4). Surprisingly only three tandem clusters were detected.

Due to the huge difference in their predicted structures, two separate phylogeny trees were built (supplementary fig. S4, Supplementary Material online): one for Types I and II proteins and one for Types III and IV. The large phylogenetic distance between the family members is mainly explained by the presence/absence of specific domains and also by the variation of their position on the sequence.

### GRAS

**Fig. 5.—:**
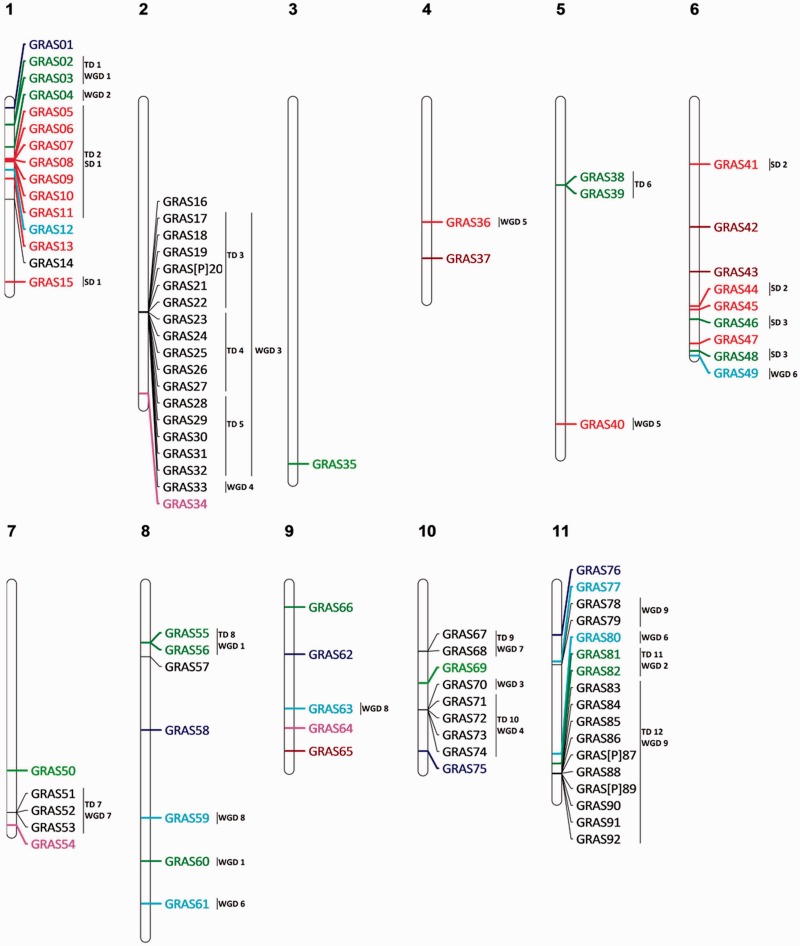
Genomic localization of GRAS genes from *E. grandis*. All the predicted GRAS genes including complete sequences, partial sequences, and pseudogenes are presented. GRAS type SHR genes are marked in blue, GRAS type HAM genes in green, GRAS type PAT1 genes in red, GRAS type SCR genes in blue fluo, GRAS type LISCL genes in black, GRAS type SCL3 genes in purple, and GRAS type LS genes in brown. TD clusters, SD events, and WGD events are displayed on the right side of the corresponding sequences or segments.

Ninety-two GRAS members have been found in the *E. grandis* genome, of which 20% required a reannotation. The family size is comparable to that of *P. trichocarpa* but is much larger than in *A. thaliana* and *V. vinifera* (table 1 and supplementary table S4, Supplementary Material online). The higher number of GRAS sequences in *E. grandis* is mainly due to TDs for PAT1 and LISCL subfamilies (38 in *E. grandis* and 6 in *A. thaliana)*, mainly located in chromosomes 1, 2, 10, and 11 (fig. 5 and supplementary fig. S6, Supplementary Material online). The role of PAT1 and LISCL in general processes such as plant development and plant defense response (Sun et al. 2012) can support the gene number explosion.

### Late Embryogenesis Abundant

**Fig. 6.—:**
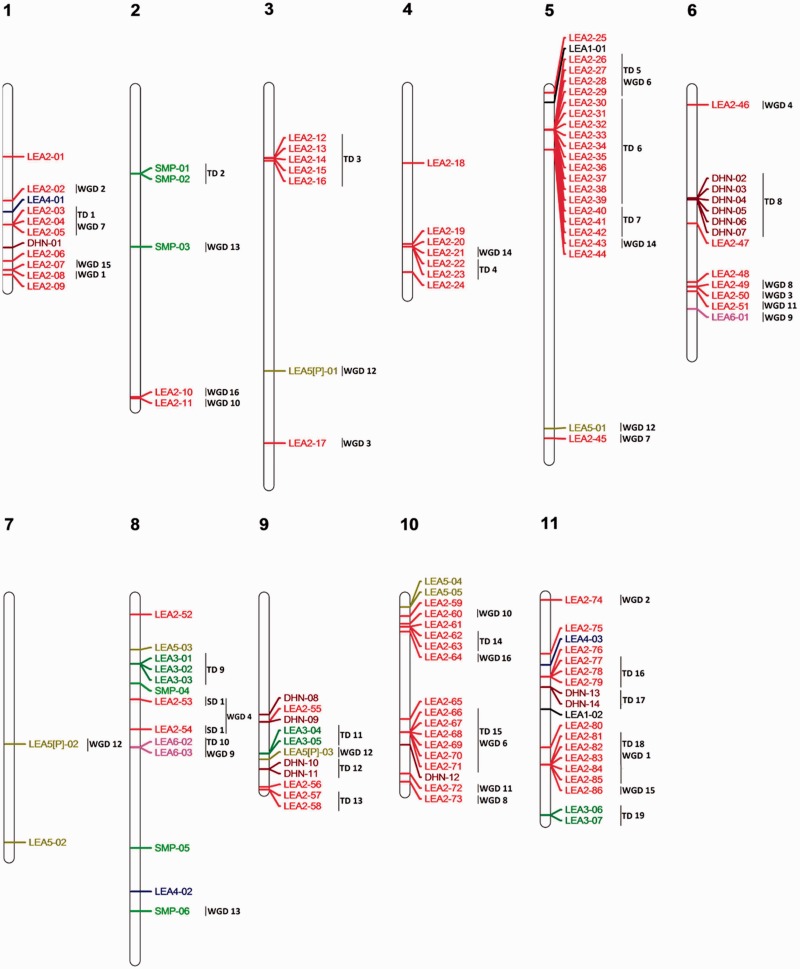
Genomic localization of LEA genes from *E. grandis*. All the predicted LEA genes including complete sequences, partial sequences, and pseudogenes are presented. LEA1 are marked in dark, LEA2 and LEA like in red, LEA3 in green, LEA4 in blue, LEA5 in pale green, LEA6 in pink, SMP in fluo green, and dehydrine (DHN) in brown. TD clusters, SD events, and WGD events are displayed on the right side of the corresponding sequences or segments.

Like for DNAj, reannotation has been done for the four organisms. One hundred twenty-nine LEAs have been found in the *E. grandis* genome which is more than in the three other species. Thirty-six percent required a reannotation (table 1 and supplementary table S5, Supplementary Material online). The analysis of the gene’s loci map showed that the LEA family members are spotty distributed on the 11 chromosomes, indicating species-specific composition of the subfamily. The explosion of LEA isoform number is mainly due to large duplication events of LEA2, sub class LEA-like, such as those involving 15 and 21 LEA-like on chromosome 10 and 5, respectively (fig. 6 and supplementary fig. S8, Supplementary Material online).

### Peroxidases Families: APx and CIII Prx

**Fig. 7.—:**
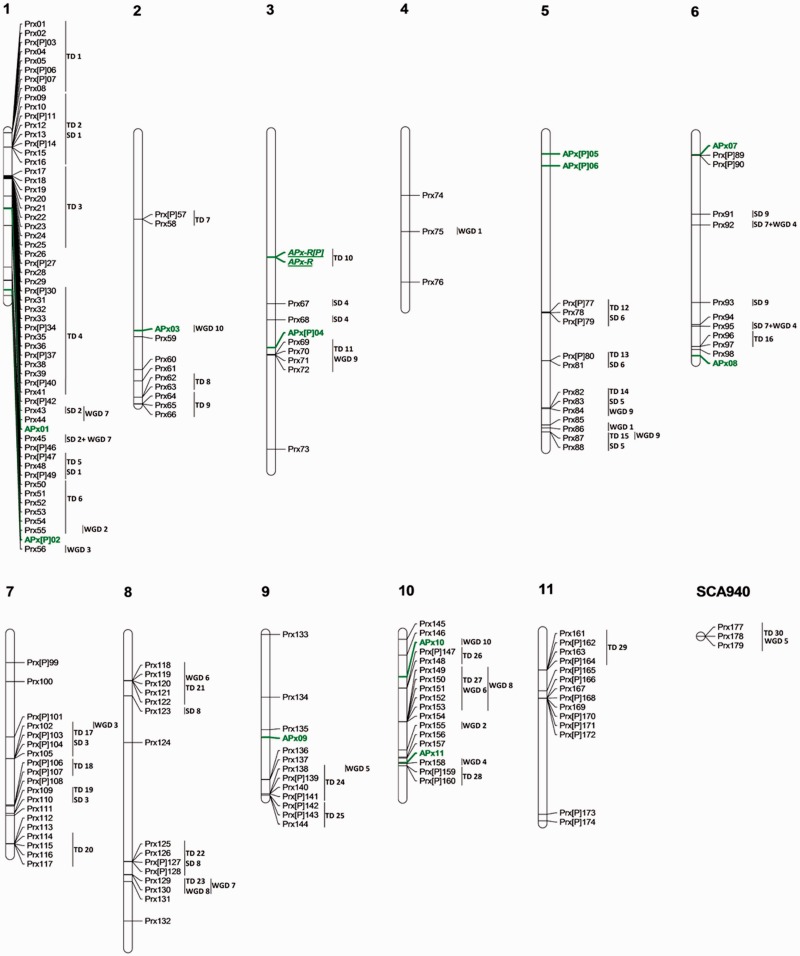
Genomic localization of Peroxidase gene family from *E. grandis*. All the predicted APx and CIII Prx genes including complete sequences, partial sequences, and pseudogenes are presented. APx genes are marked in green including APx-R which are in italic and underlined. CIII Prx genes are marked in black. TD clusters, SD events, and WGD events are displayed on the right side of the corresponding sequences or segments.

Thirteen APx sequences and 181 CIII Prx sequences have been annotated in *E. grandis* where 65% required a reannotation (supplementary table S6, Supplementary Material online). As expected, the APxs present no significant variation of isoform numbers between organisms (table 1). However, CIII Prx number is the highest among dicotyledons due to 30 TDs and 8 SDs with a remarkable concentration of sequences on chromosome 1, where 56 CIII Prxs have been detected (fig. 7).

The phylogenetic tree of CIII Prxs allows identifying five main clusters of CIII Prxs (I–V) with large species-specific clusters (supplementary fig. S10, Supplementary Material online).

## Discussion

Although the quality of the annotations of new genomes has been improved, the percentage of incorrect or missing annotations remains high. For most of the families annotated, the number of proteins extracted from the predicted proteome contains several theoretical alternative transcripts of the same gene, partial sequences and did not contain the whole number of isoforms. Recent duplications, source of gene clusters, are often misannotated. Therefore, it appears necessary to obtain exhaustive and of high quality sets of proteins for a phylogenetic analysis. The protocol that combines automatic and expert annotation is time consuming but allows the reduction of the number of mispredictions and increases the coverage of the annotation. The correct reannotation (partial and pseudogene sequences, fused or not predicted Open reading frame [ORF]), is necessary because it changes the evolutionary conclusions made from global family analyses.

Through the analysis of eight multigenic families, two evolutionary situations are observed. First, the number of paralogs remains stable from one organism to another. This is the case of DNAj, APx, and AUX/LAX. These proteins are therefore not subjected to recent evolution because few TDs are observed in the various phylogenetic analyses (table 3). In addition, no aborted duplication events are observed because no pseudogenes were detected during the exhaustive data mining. The lack of variation of the isoform numbers between species together with the strong conservation between orthologs may suggest a negative selection regarding the importance of the protein function. The implication of some of these proteins in protein complexes such as DNAj with HSP70 also justifies the gene number conservation.

**Table 3:** Duplication Events Detected in *Eucalyptus grandis* Genome Based on Paralog/Ortholog Relationship and Chromosomal Localization

Family	TD Clusters	TD Events	SD Events	WGD Events
**AP2/ERF**	17	62 (39%)	14	19
**GRAS**	10	40 (54%)	3	9
**PIN**	2	2 (24%)	2	1
**AUX/LAX**	0	0	0	0
**APx**	1	1 (15%)	0	1
**CIII Prx**	28	59 (48%)	8	10
**LEA**	19	47 (51%)	1	14
**DNAj/HSP40**	5	5 (5%)	6	19

note.—The numbers of TD clusters, TD events, SD events, and WGD events were listed in this table. Such as TD clusters can be composed with more than two tandemly duplicated genes, the total TD events corresponded with the number of duplicated genes minus the number of TD clusters. The percentage of genes implicated in TDs is notified in brackets (*n*/family size).

On the other hand, the significant increase in family size observed when comparing the four species under study is mainly due to the high number of isoforms of some classes (or subfamilies) such as DREB and ERF for AP2/ERF family; the cluster II.3 and 4 for CIII Prx; EgrPIN group II, PAT1, and LISCL for GRAS family and LEA2. The increase in isoform numbers is mainly due to TDs while some SDs led to a large cluster of paralogs in restricted areas. Even if, in some cases, these large clusters contain pseudogenes reflecting the disappearance of some of the duplicated genes, the majority of the paralogs is conserved suggesting a positive selection. These duplication events and retention of paralogs can be somewhat advantageous for *E. grandis* and could lead to either sub- and neofunctionalization or to a dosage effect. To support this hypothesis, some duplicated sequences present different expression profiles such as *DREB03* to *17* (supplementary fig. S2, Supplementary Material online), DNAj05 and 06 (supplementary fig. S5, Supplementary Material online), and GRAS (TDs 3–5 and 12, supplementary fig. S7, Supplementary Material online) or similar expression profiles such as LEA-like (TDs 18, supplementary fig. S9, Supplementary Material online) and CIII Prxs (TDs 2–-23 and 29, supplementary fig. S11, Supplementary Material online).

The frequency of duplication events appeared to be connected to the size of the family analyzed. Except for the PIN family, these gene expansion events are mainly observed within large multigenic families therefore more statistically prone to duplication. The significant variation of the gene number together with the conservation of these duplication events suggest a selective pressure leading to diversifying outcomes. It could be related with the *E. grandis* morphological and physiological characteristics such as growth rate or nondormancy capacity. Functional and expression analysis of these duplicated genes could further confirm this hypothesis.

**Fig. 8.—:**
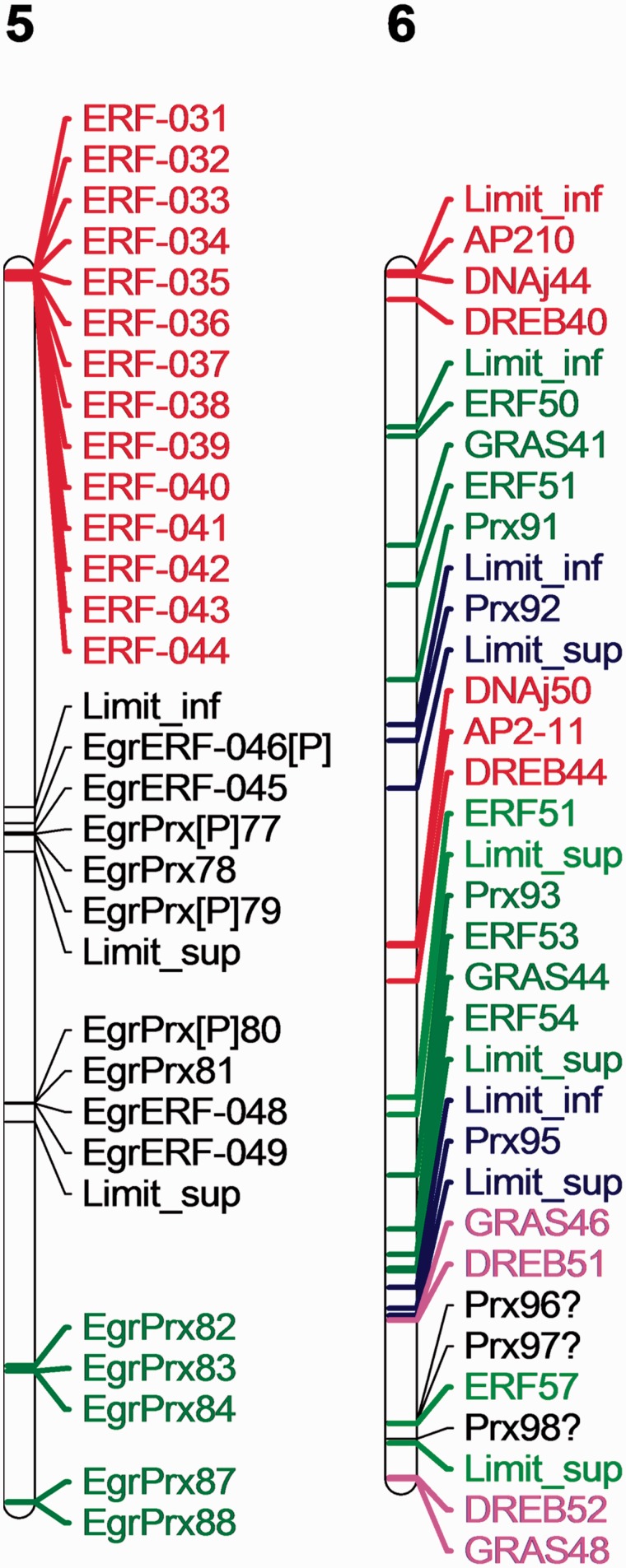
Illustration of SD observed in the chromosome 5 and 6. AP2/ERF genes, CIII Prx genes, and DNAj gene part of duplications localized on chromosomes 5 and 6. Same color corresponds to the two parts of SD. Genes with question mark are missing from one duplicated segment. Size of duplicated segment has been increased when data were available from the *Eucalyptus* consortium and noted limit_sup or limit_inf. This synthetic chromosomal localization is displayed by MapChart 2.1.

In a general manner, SDs and WGDs are detected regardless of the evolutionary situations and are not significantly different between two protein groups of similar size even if their size increases relatively to that of *A. thaliana*. In contrast, the number of TDs is very high in the case of a protein family whose size increases relatively to that of *A. thaliana* (tables 1 and 3). Regarding the gene distribution, hot spots of TDs combined with SDs are detected in chromosomes 1, 2, 5, 6, 7, and 10. On the other hand, the other chromosomes (3, 4, 8, and 9) contain fewer duplication events. The complexity of the duplication events is illustrated for the chromosome 5 and 6 where several SDs with internal rearrangements and TDs were detected (fig. 8). It appears that sequence (function/role) and chromosomal location can be correlated with these hot spots. For example, the duplications of DREB genes, described as regulators of abiotic stress responses mainly located in the chromosome 1, could be necessary for *E. grandis* to cope with various environmental changes. Similarly, a cluster of GRAS and another of CIII Prx proteins, with roles for growth and plant defense response, are mainly located in chromosome 1. In this case, hot spots leading to numerous paralogs may restore the correct dosage balance in a dosage sensitive system.

Nevertheless many questions are still unsolved and need further investigation to be correctly addressed, such as: why are some families (clusters or subclasses) subjected to numerous duplication events while other protein families have kept a similar gene number after speciation? Do gene functions promote/control gene duplication? And are these duplications associated with their chromosomal locations?

## Supplementary Material

Supplementary files S1 and S2 are available at *Genome Biology and Evolution* online (http://www.gbe.oxfordjournals.org/).

Supplementary Data
