# Supplementary material for: Explosive Tandem and Segmental Duplications of Multigenic Families in Eucalyptus grandis
Source: Genome Biol Evol. 2015 Mar 13;7(4):1068–81. doi: 10.1093/gbe/evv048 (PMC4419795; doi:10.1093/gbe/evv048)
Supplement: Supplementary Data [file supp_7_4_1068__index.html]

Explosive Tandem and Segmental Duplications of Multigenic Families in Eucalyptus grandis — Supplementary Data 

# Explosive Tandem and Segmental Duplications of Multigenic Families in *Eucalyptus grandis*

## Supplementary Data

files

**Files in this Data Supplement:**

- Supplementary Data - pdf file
- Supplementary Data - docx file
- Supplementary Data - xls file
