# Supplementary material for: Explosive Tandem and Segmental Duplications of Multigenic Families in Eucalyptus grandis
Source: Genome Biol Evol. 2015 Mar 13;7(4):1068–81. doi: 10.1093/gbe/evv048 (PMC4419795; doi:10.1093/gbe/evv048)

Supplementary_Files_S1_Figures S1-S16

Fig S1 Phylogenetic representation of AP2 proteins from *E. grandis* and *A. thaliana*.

Fig S2 Heat map of the expression of the AP2/ERF genes from *E. grandis* in seven different tissues determined by RNA-seq.

Fig S3 Phylogenetic representation of auxin transporters PIN (A) and AUX/LAX (B) proteins from *E. grandis* and *A. thaliana*.

Fig S4 Phylogenetic representation of DNAj proteins from *E. grandis* and *A. thaliana.*

Fig S5 Heat map of the expression of the DNAj genes from *E. grandis* in seven different tissues determined by RNA-seq.

Fig S6 Phylogenetic representation of GRAS proteins from *E. grandis* and *A. thaliana.*

Fig S7 Heat map of the expression of the GRAS genes from *E. grandis* in seven different tissues determined by RNA-seq.

Fig S8 Phylogenetic representation of LEA proteins from *E. grandis* and *A. thaliana.*

Fig S9 Heat map of the expression of the LEA genes from *E. grandis* in seven different tissues determined by RNA-seq.

Fig S10 Phylogenetic representation of peroxidase family: APx and CIII Prx proteins from *E. grandis* and *A. thaliana.*

Fig S11 Heat map of the expression of the APx and CIII Prx genes from *E. grandis* in seven different tissues determined by RNA-seq.

Fig. S1 Phylogenetic representation of AP2 proteins from *E. grandis* and *A. thaliana*. (a) sub-family AP2; (b) sub-family RAV; (c) sub-family DREB; (d) sub-family ERF. The bootstraps were written on the trees. The clustering of sub-family DREB and ERF were analyzed and marked with vertical lines and cluster (sub-cluster) names in different colors in (c) and (d).

(a)

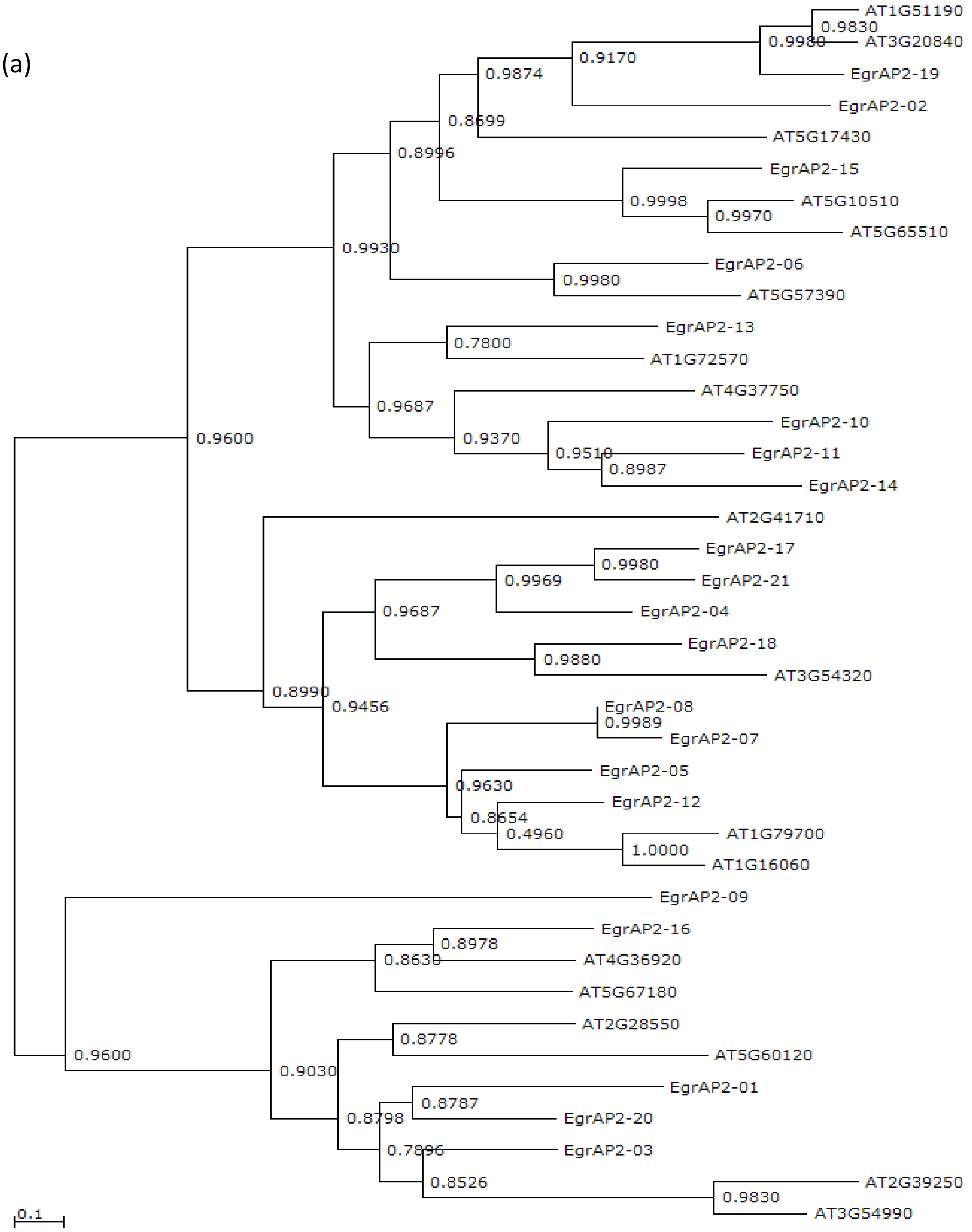

AT1G51190
0.9830
0.9980
AT3G20840
EgrAP2-19
0.9170
EgrAP2-02
0.9874
AT5G17430
0.8699
0.8996
EgrAP2-15
0.9998
AT5G10510
0.9970
AT5G65510
0.9930
EgrAP2-06
0.9980
AT5G57390
EgrAP2-13
0.7800
AT1G72570
0.9687
AT4G37750
EgrAP2-10
0.9370
0.9510
EgrAP2-11
0.8987
EgrAP2-14
0.9600
AT2G41710
EgrAP2-17
0.9980
EgrAP2-21
0.9969
EgrAP2-04
0.9687
EgrAP2-18
0.9880
AT3G54320
0.8990
0.9456
EgrAP2-08
0.9989
EgrAP2-07
EgrAP2-05
0.9630
EgrAP2-12
0.8654
0.4960
AT1G79700
1.0000
AT1G16060
EgrAP2-09
EgrAP2-16
0.8978
0.8630
AT4G36920
AT5G67180
AT2G28550
0.8778
0.9600
AT5G60120
0.9030
EgrAP2-01
0.8787
0.8798
EgrAP2-20
0.7896
EgrAP2-03
0.8526
AT2G39250
0.9830
AT3G54990
0.1

(b)

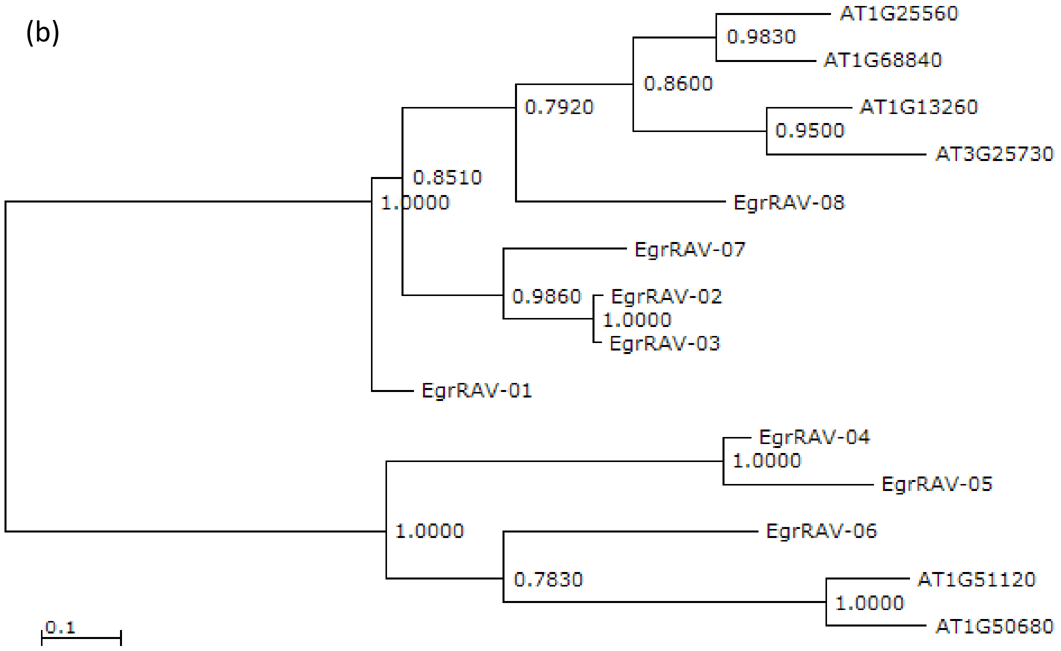
AT1G25560
0.9830
AT1G68840
0.8600
0.7920
AT1G13260
0.9500
AT3G25730
0.8510
1.0000
EgrRAV-08
EgrRAV-07
0.9860
EgrRAV-02
1.0000
EgrRAV-03
EgrRAV-01
EgrRAV-04
1.0000
EgrRAV-05
1.0000
EgrRAV-06
0.7830
AT1G51120
1.0000
AT1G50680
0.1

(c)

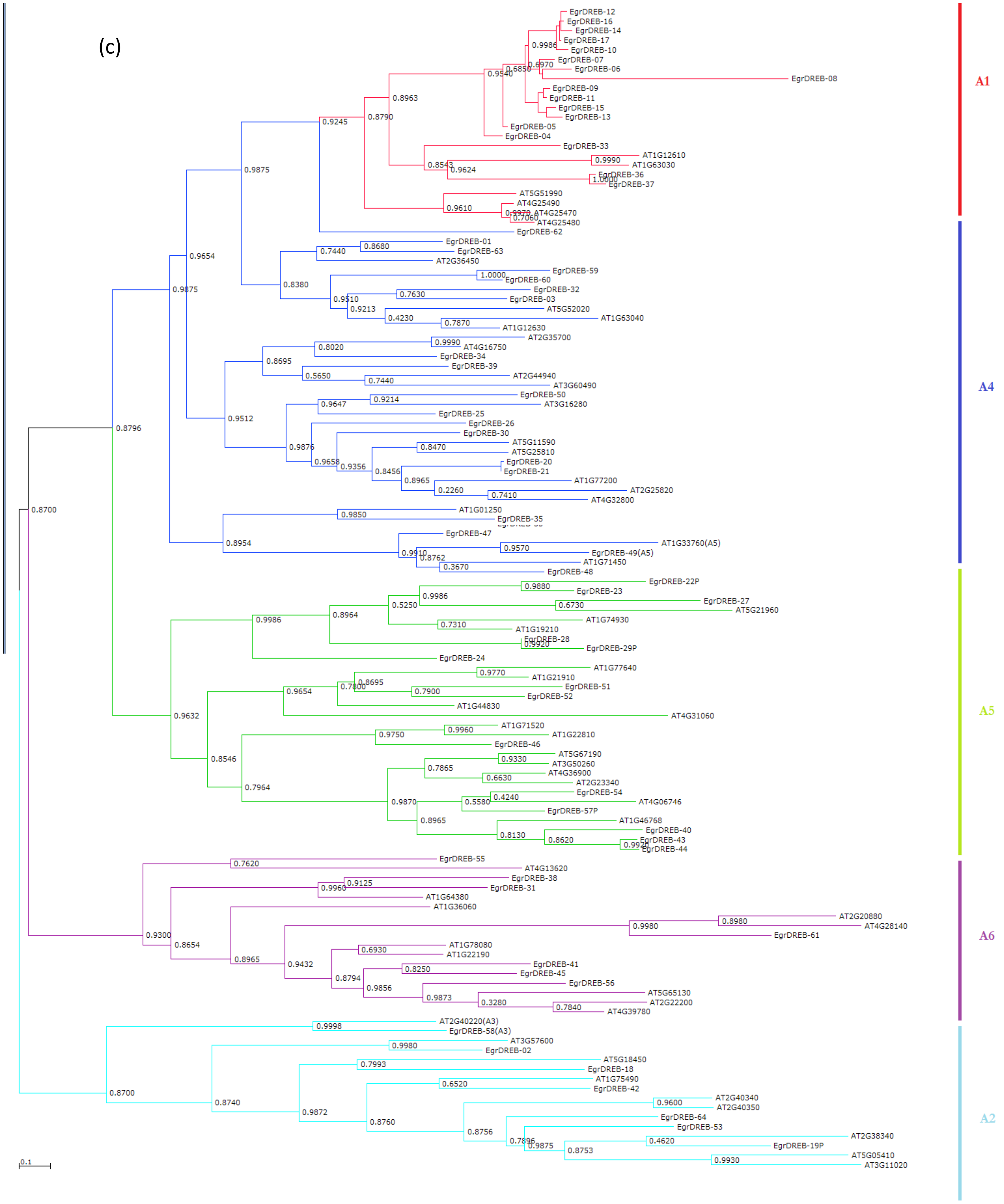
EgrDREB-12
EgrDREB-16
EgrDREB-14
EgrDREB-17
EgrDREB-10
EgrDREB-07
EgrDREB-06
EgrDREB-08
EgrDREB-09
EgrDREB-11
EgrDREB-15
EgrDREB-13
EgrDREB-05
EgrDREB-04
EgrDREB-33
AT1G12610
AT1G63030
EgrDREB-36
EgrDREB-37
AT5G51990
AT4G25490
AT4G25470
AT4G25480
EgrDREB-62
EgrDREB-01
EgrDREB-63
AT2G36450
EgrDREB-59
EgrDREB-60
EgrDREB-32
EgrDREB-03
AT5G52020
AT1G63040
AT1G12630
AT2G35700
AT4G16750
EgrDREB-34
EgrDREB-39
AT2G44940
AT3G60490
EgrDREB-50
AT3G16280
EgrDREB-25
EgrDREB-26
EgrDREB-30
AT5G11590
AT5G25810
EgrDREB-20
EgrDREB-21
AT1G77200
AT2G25820
AT4G32800
AT1G01250
EgrDREB-35
EgrDREB-47
AT1G33760(A5)
EgrDREB-49(A5)
AT1G71450
EgrDREB-48
EgrDREB-22P
EgrDREB-23
EgrDREB-27
AT5G21960
AT1G74930
AT1G19210
EgrDREB-28
EgrDREB-29P
EgrDREB-24
AT1G77640
AT1G21910
EgrDREB-51
EgrDREB-52
AT1G44830
AT4G31060
AT1G71520
AT1G22810
EgrDREB-46
AT5G67190
AT3G50260
AT4G36900
AT2G23340
EgrDREB-54
AT4G06746
EgrDREB-57P
AT1G46768
EgrDREB-40
EgrDREB-43
EgrDREB-44
EgrDREB-55
AT4G13620
EgrDREB-38
EgrDREB-31
AT1G64380
AT1G36060
AT2G20880
AT4G28140
EgrDREB-61
AT1G78080
AT1G22190
EgrDREB-41
EgrDREB-45
EgrDREB-56
AT5G65130
AT2G22200
AT4G39780
AT2G40220(A3)
EgrDREB-58(A3)
AT3G57600
EgrDREB-02
AT5G18450
EgrDREB-18
AT1G75490
EgrDREB-42
AT2G40340
AT2G40350
EgrDREB-64
EgrDREB-53
AT2G38340
EgrDREB-19P
AT5G05410
AT3G11020
0.1
A1
A4
A5
A6
A2

(d)

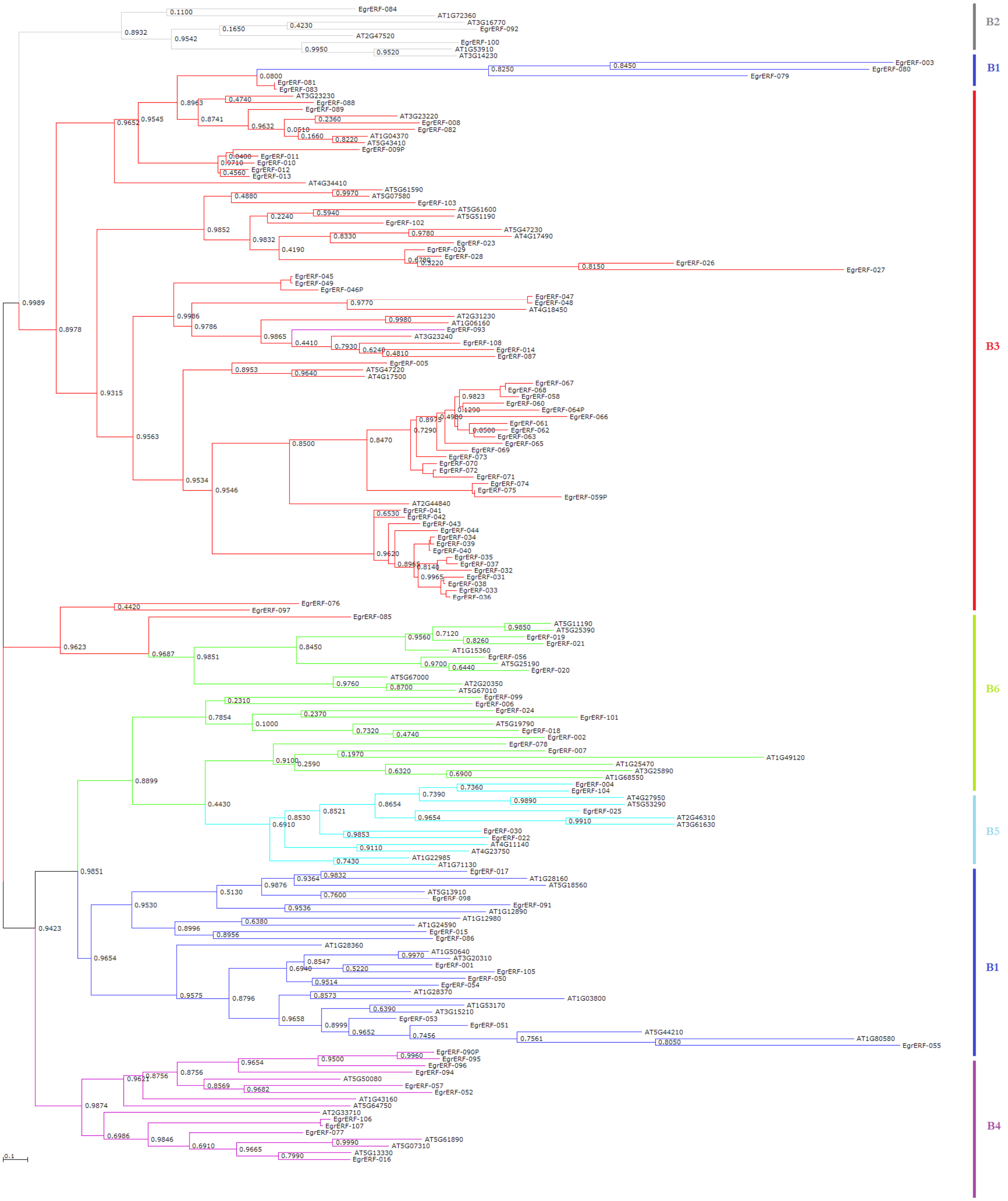

EgrERF-084
AT1G72360
AT3G16770
EgrERF-092
AT2G47520
EgrERF-100
AT1G53910
AT3G14230
EgrERF-003
EgrERF-080
EgrERF-079
EgrERF-081
EgrERF-083
AT3G23230
EgrERF-088
EgrERF-089
AT3G23220
EgrERF-008
EgrERF-082
AT1G04370
AT5G43410
EgrERF-009P
EgrERF-011
EgrERF-010
EgrERF-012
EgrERF-013
AT4G34410
AT5G61590
AT5G07580
EgrERF-103
AT5G61600
AT5G51190
EgrERF-102
AT5G47230
AT4G17490
EgrERF-023
EgrERF-029
EgrERF-028
EgrERF-026
EgrERF-027
EgrERF-045
EgrERF-049
EgrERF-046P
EgrERF-047
EgrERF-048
AT4G18450
AT2G31230
AT1G06160
EgrERF-093
AT3G23240
EgrERF-108
EgrERF-014
EgrERF-087
EgrERF-005
AT5G47220
AT4G17500
EgrERF-067
EgrERF-068
EgrERF-058
EgrERF-060
EgrERF-064P
EgrERF-066
EgrERF-061
EgrERF-062
EgrERF-063
EgrERF-065
EgrERF-069
EgrERF-073
EgrERF-070
EgrERF-072
EgrERF-071
EgrERF-074
EgrERF-075
EgrERF-059P
AT2G44840
EgrERF-041
EgrERF-042
EgrERF-043
EgrERF-044
EgrERF-034
EgrERF-039
EgrERF-040
EgrERF-035
EgrERF-037
EgrERF-032
EgrERF-031
EgrERF-038
EgrERF-033
EgrERF-036
EgrERF-076
EgrERF-097
EgrERF-085
AT5G11190
AT5G25390
EgrERF-019
EgrERF-021
AT1G15360
EgrERF-056
AT5G25190
EgrERF-020
AT5G67000
AT2G20350
AT5G67010
EgrERF-099
EgrERF-006
EgrERF-024
EgrERF-101
AT5G19790
EgrERF-018
EgrERF-002
EgrERF-078
EgrERF-007
AT1G49120
AT1G25470
AT3G25890
AT1G68550
EgrERF-004
EgrERF-104
AT4G27950
AT5G53290
EgrERF-025
AT2G46310
AT3G61630
EgrERF-030
EgrERF-022
AT4G11140
AT4G23750
AT1G22985
AT1G71130
EgrERF-017
AT1G28160
AT5G18560
AT5G13910
EgrERF-098
EgrERF-091
AT1G12890
AT1G12980
AT1G24590
EgrERF-015
EgrERF-086
AT1G28360
AT1G50640
AT3G20310
EgrERF-001
EgrERF-105
EgrERF-050
EgrERF-054
AT1G28370
AT1G03800
AT1G53170
AT3G15210
EgrERF-053
EgrERF-051
AT5G44210
AT1G80580
EgrERF-055
EgrERF-090P
EgrERF-095
EgrERF-096
EgrERF-094
AT5G50080
EgrERF-057
EgrERF-052
AT1G43160
AT5G64750
AT2G33710
EgrERF-106
EgrERF-107
EgrERF-077
AT5G61890
AT5G07310
AT5G13330
EgrERF-016
0.1
B2
B1
B3
B6
B5
B1
B4

Fig. S2 Heat map of the expression of the AP2/ERF genes from *E. grandis* in seven different tissues determined by RNA-seq. The chromosomal localization of each gene was listed on the right.

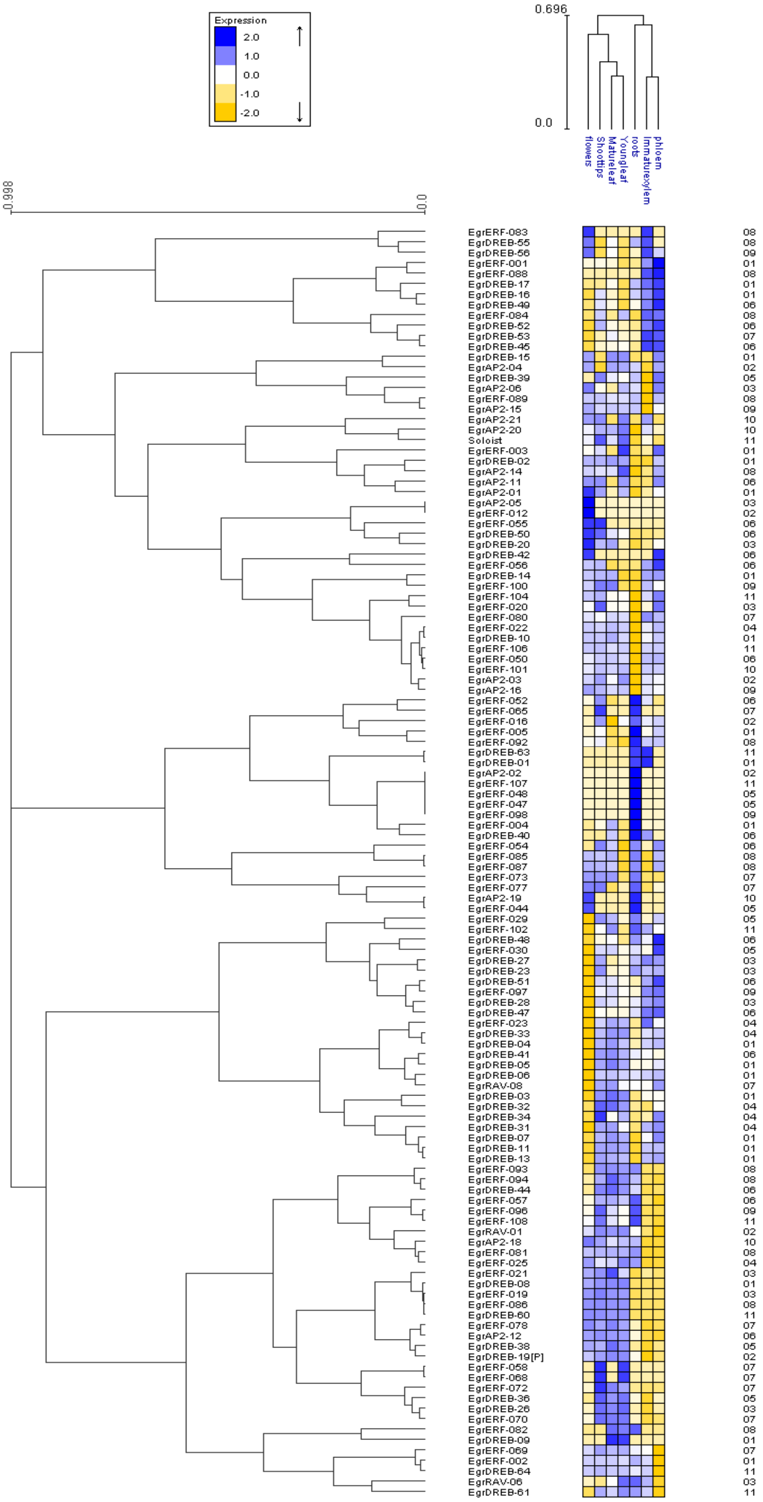

Fig. S3 Phylogenetic representation of auxin transporters PIN (a) and AUX/LAX (b) proteins from *E. grandis* and *A. thaliana*. The bootstraps were written on the trees. The intron numbers and the chromosomal localization of each gene were listed on the right of the trees. The gene structures were also visualized on the right sides. The clustering of family PIN was analyzed and marked with vertical lines and cluster (sub-cluster) names in different colors in (a).

(a)

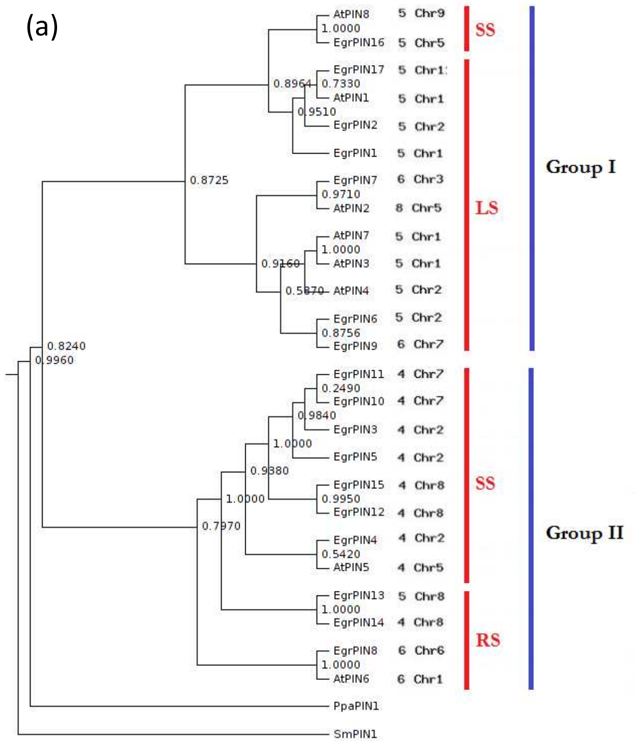

(b)

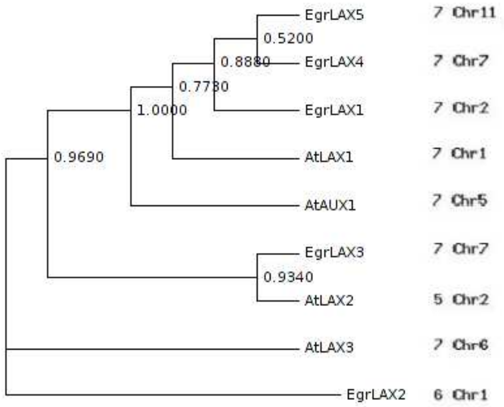

Fig. S4 Phylogenetic representation of DNAj proteins from *E. grandis* and *A. thaliana*. Schematic radial phylogeny (a) and horizontal cladogram and (b) of the whole set of DNAj. The bootstraps were written on the tree nodes.

(a)

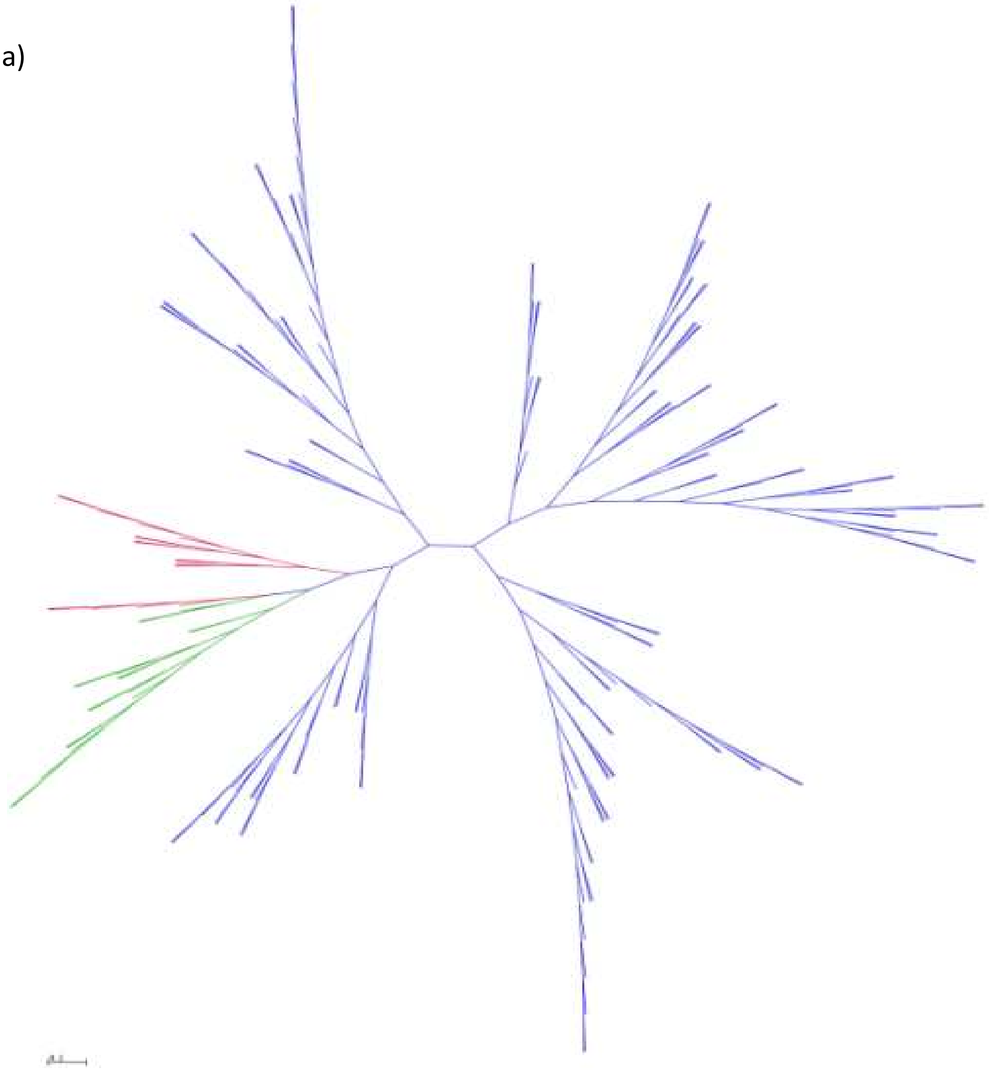

(b)

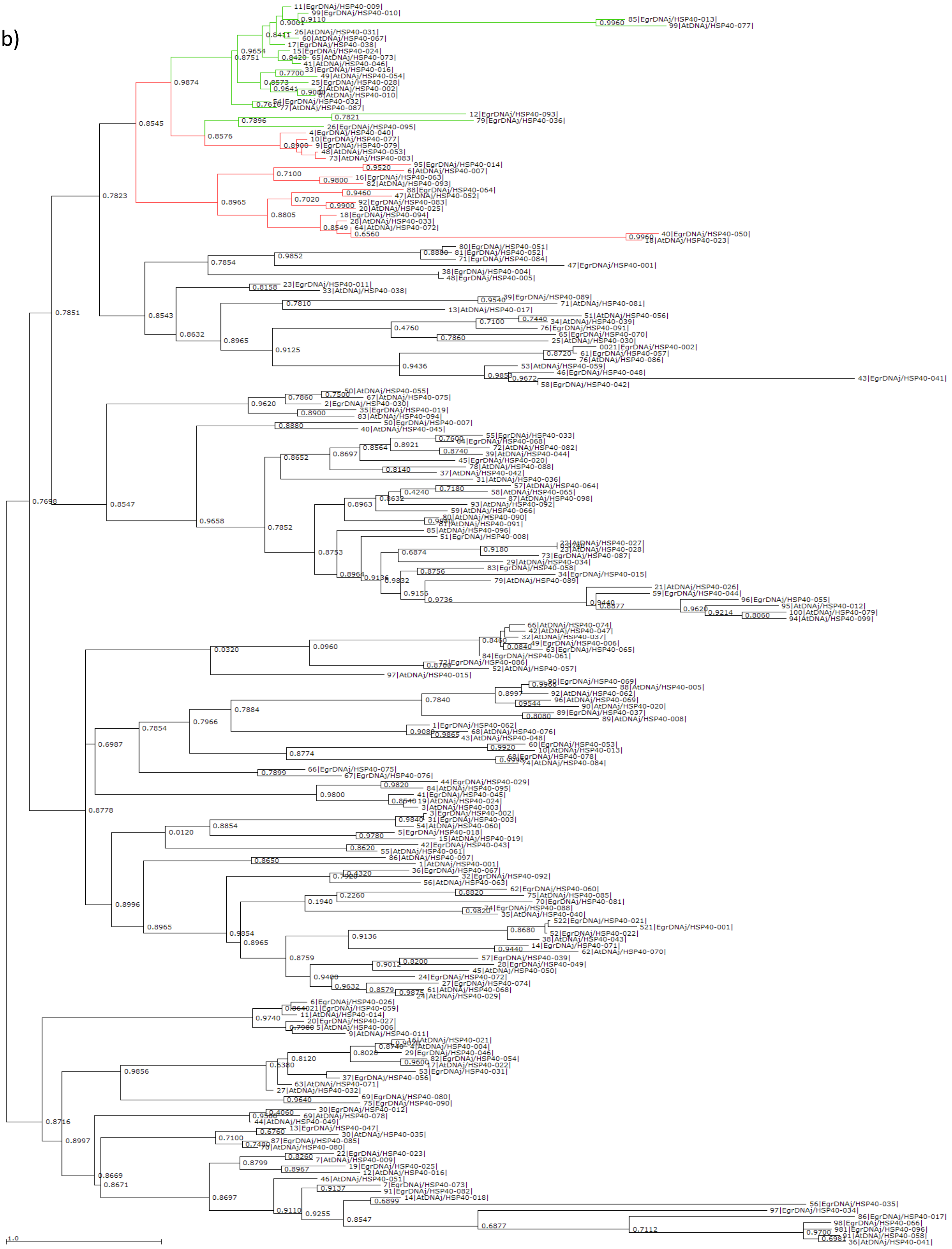

Fig. S5 Heat map of the expression of the DNAj genes from *E. grandis* in seven different tissues determined by RNA-seq. The chromosomal localization of each gene was listed on the right.

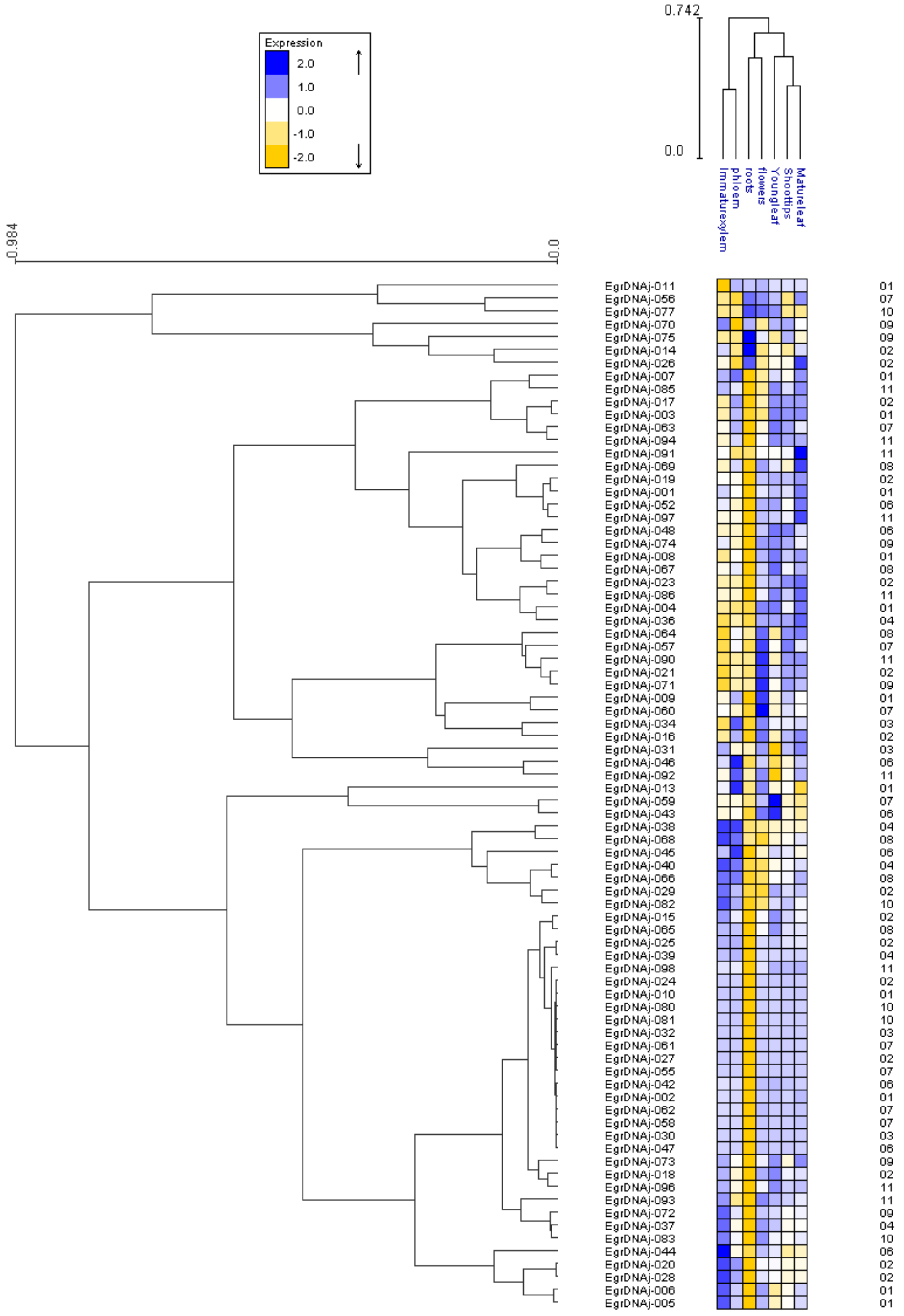

Fig. S6 Phylogenetic representation of GRAS proteins from *E. grandis* and *A. thaliana*. The bootstraps were written on the tree nodes.

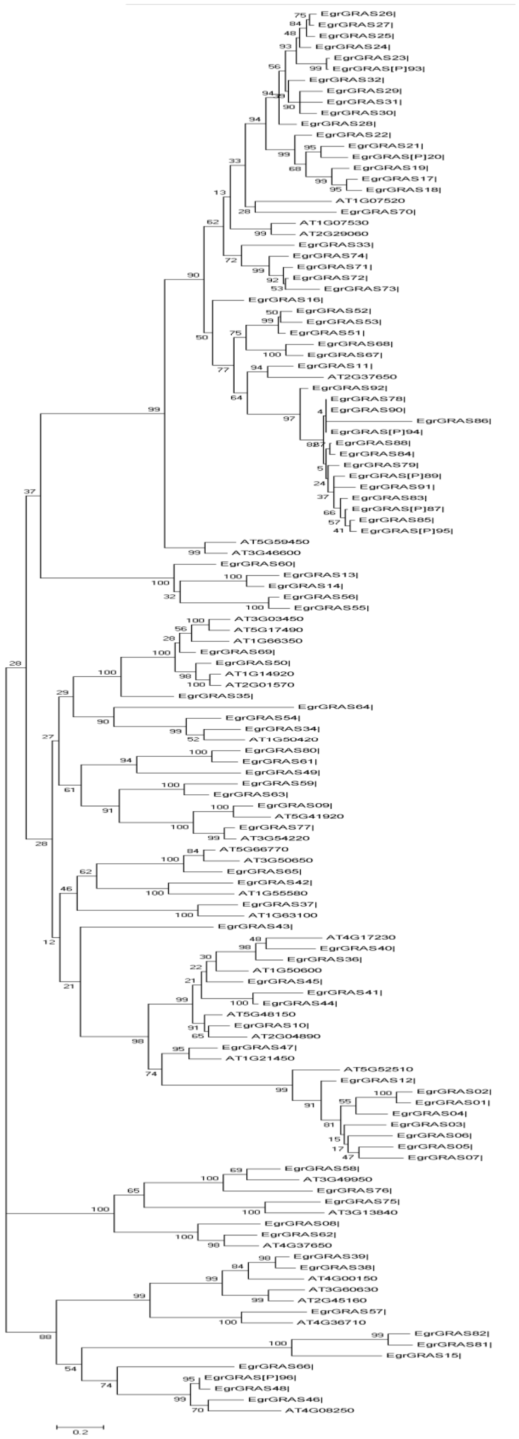

Fig. S7 Heat map of the expression of the GRAS genes from *E. grandis* in seven different tissues determined by RNA-seq. The chromosomal localization of each gene was listed on the right.

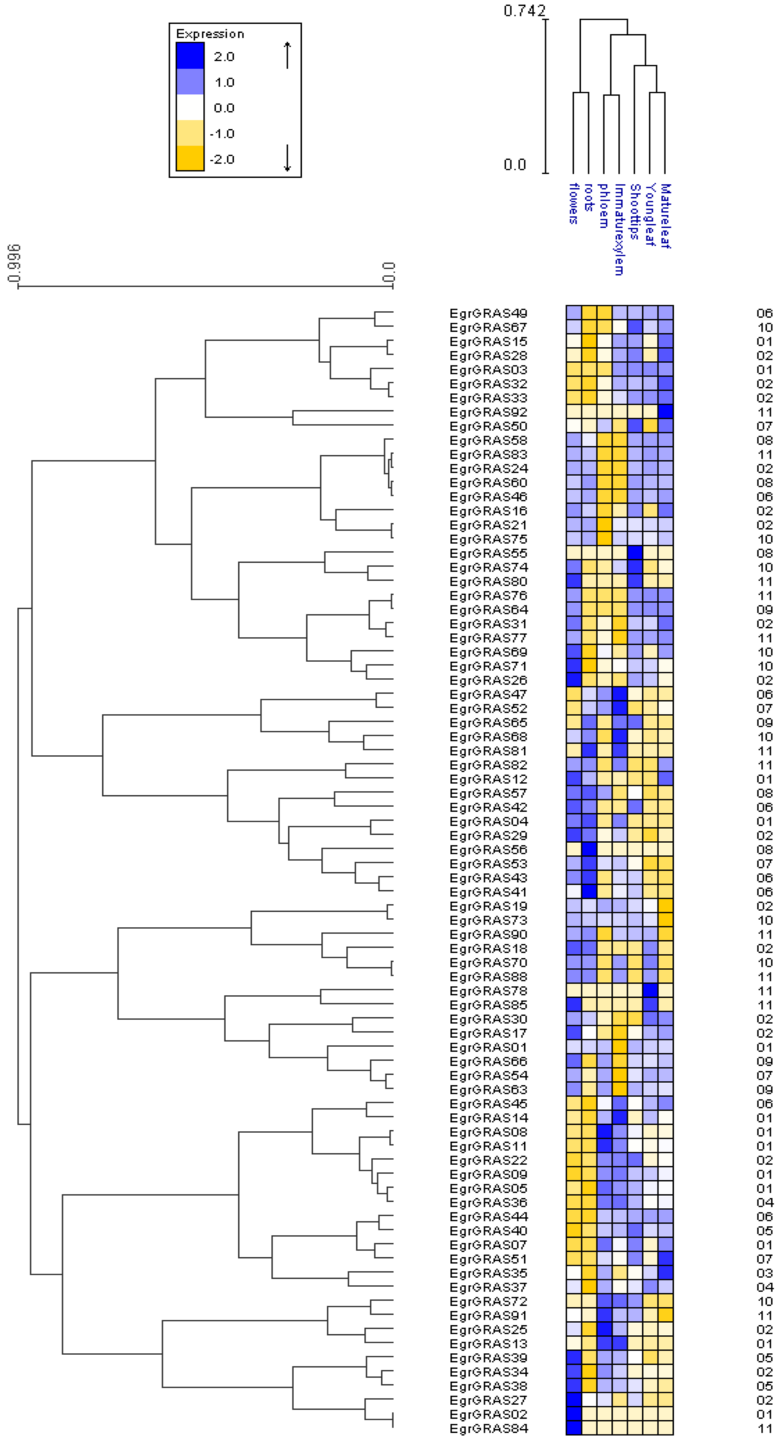

Fig. S8 Phylogenetic representation of LEA proteins from *E. grandis* and *A. thaliana*. A schematic radial phylogeny of the whole set of LEA (a) and its horizontal cladogram (b). The LEA2 family is represented in dark blue, LEA5 in azure, LEA6 in violet, LEA/DNH in orange, LEA/SMP in pink, LEA1 in green, LEA3 in red and the common root in black. The bootstraps were written on the tree nodes (b).

(a)

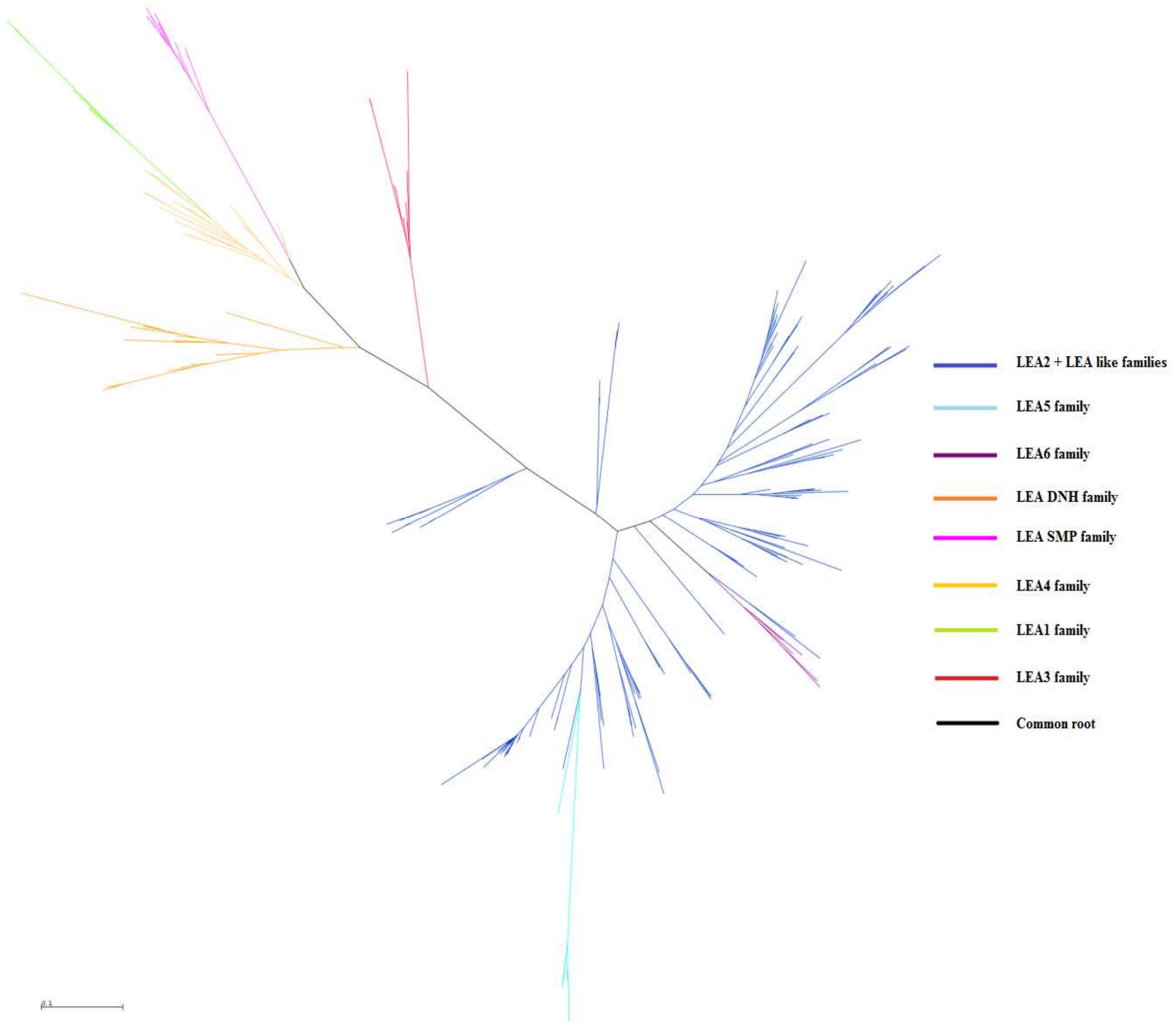

(b)

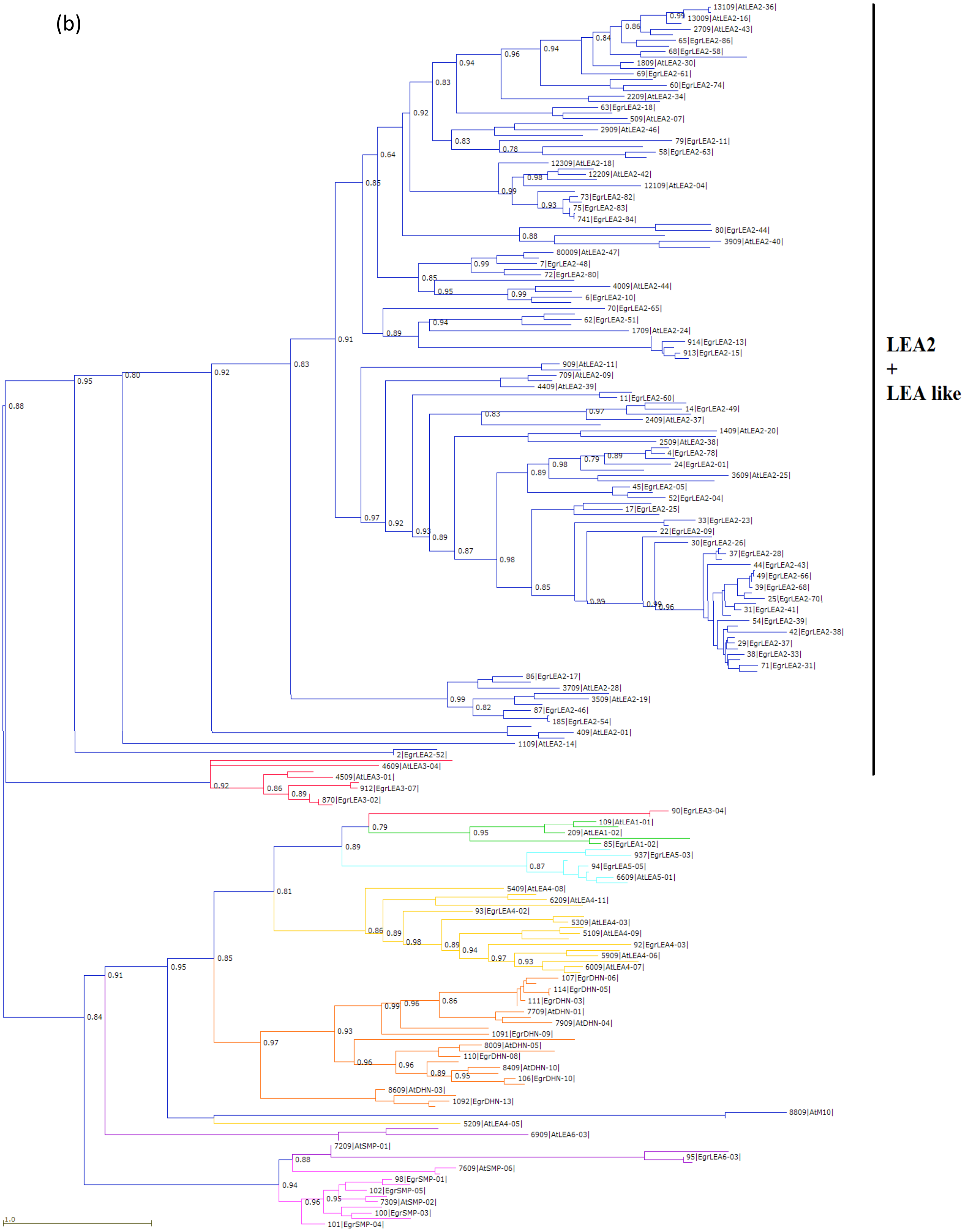
13109|AtLEA2-36|
13009|AtLEA2-16|
2709|AtLEA2-43|
65|EgrLEA2-86|
68|EgrLEA2-58|
1809|AtLEA2-30|
69|EgrLEA2-61|
60|EgrLEA2-74|
2209|AtLEA2-34|
63|EgrLEA2-18|
509|AtLEA2-07|
2909|AtLEA2-46|
79|EgrLEA2-11|
58|EgrLEA2-63|
12309|AtLEA2-18|
12209|AtLEA2-42|
12109|AtLEA2-04|
73|EgrLEA2-82|
75|EgrLEA2-83|
741|EgrLEA2-84|
80|EgrLEA2-44|
3909|AtLEA2-40|
80009|AtLEA2-47|
7|EgrLEA2-48|
72|EgrLEA2-80|
4009|AtLEA2-44|
6|EgrLEA2-10|
70|EgrLEA2-65|
62|EgrLEA2-51|
1709|AtLEA2-24|
914|EgrLEA2-13|
913|EgrLEA2-15|
909|AtLEA2-11|
709|AtLEA2-09|
4409|AtLEA2-39|
11|EgrLEA2-60|
14|EgrLEA2-49|
2409|AtLEA2-37|
1409|AtLEA2-20|
2509|AtLEA2-38|
4|EgrLEA2-78|
24|EgrLEA2-01|
3609|AtLEA2-25|
45|EgrLEA2-05|
52|EgrLEA2-04|
17|EgrLEA2-25|
33|EgrLEA2-23|
22|EgrLEA2-09|
30|EgrLEA2-26|
37|EgrLEA2-28|
44|EgrLEA2-43|
49|EgrLEA2-66|
39|EgrLEA2-68|
25|EgrLEA2-70|
31|EgrLEA2-41|
54|EgrLEA2-39|
42|EgrLEA2-38|
29|EgrLEA2-37|
38|EgrLEA2-33|
71|EgrLEA2-31|
86|EgrLEA2-17|
3709|AtLEA2-28|
3509|AtLEA2-19|
87|EgrLEA2-46|
185|EgrLEA2-54|
409|AtLEA2-01|
1109|AtLEA2-14|
2|EgrLEA2-52|
4609|AtLEA3-04|
4509|AtLEA3-01|
912|EgrLEA3-07|
870|EgrLEA3-02|
90|EgrLEA3-04|
109|AtLEA1-01|
209|AtLEA1-02|
85|EgrLEA1-02|
937|EgrLEA5-03|
94|EgrLEA5-05|
6609|AtLEA5-01|
5409|AtLEA4-08|
6209|AtLEA4-11|
93|EgrLEA4-02|
5309|AtLEA4-03|
5109|AtLEA4-09|
92|EgrLEA4-03|
5909|AtLEA4-06|
6009|AtLEA4-07|
107|EgrDHN-06|
114|EgrDHN-05|
111|EgrDHN-03|
7709|AtDHN-01|
7909|AtDHN-04|
1091|EgrDHN-09|
8009|AtDHN-05|
110|EgrDHN-08|
8409|AtDHN-10|
106|EgrDHN-10|
8609|AtDHN-03|
1092|EgrDHN-13|
8809|AtM10|
5209|AtLEA4-05|
6909|AtLEA6-03|
7209|AtSMP-01|
95|EgrLEA6-03|
7609|AtSMP-06|
98|EgrSMP-01|
102|EgrSMP-05|
7309|AtSMP-02|
100|EgrSMP-03|
101|EgrSMP-04|
1.0
LEA2
+
LEA like

Fig. S9 Heat map of the expression of the LEA genes from *E. grandis* in seven different tissues determined by RNA-seq. The chromosomal localization of each gene was listed on the right.

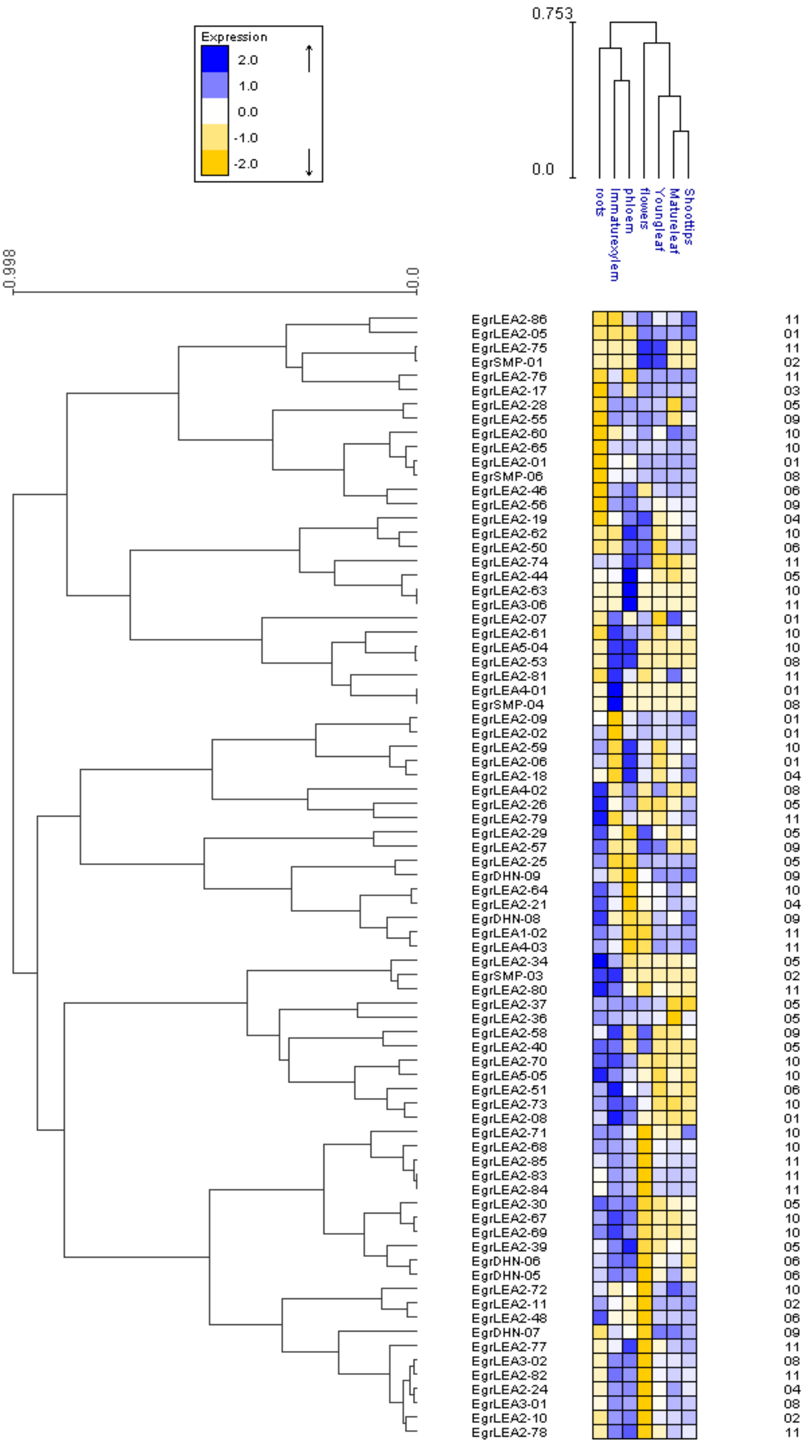

Fig. S10 Phylogenetic representation of peroxidase family: APx and CIII Prx proteins from *E. grandis* and *A. thaliana*. The chromosome localization and the intron number of each gene were written on the right of the tree. The clustering of APx and CIII Prx families were analyzed and marked with vertical lines and cluster (sub-cluster) names in different colors.

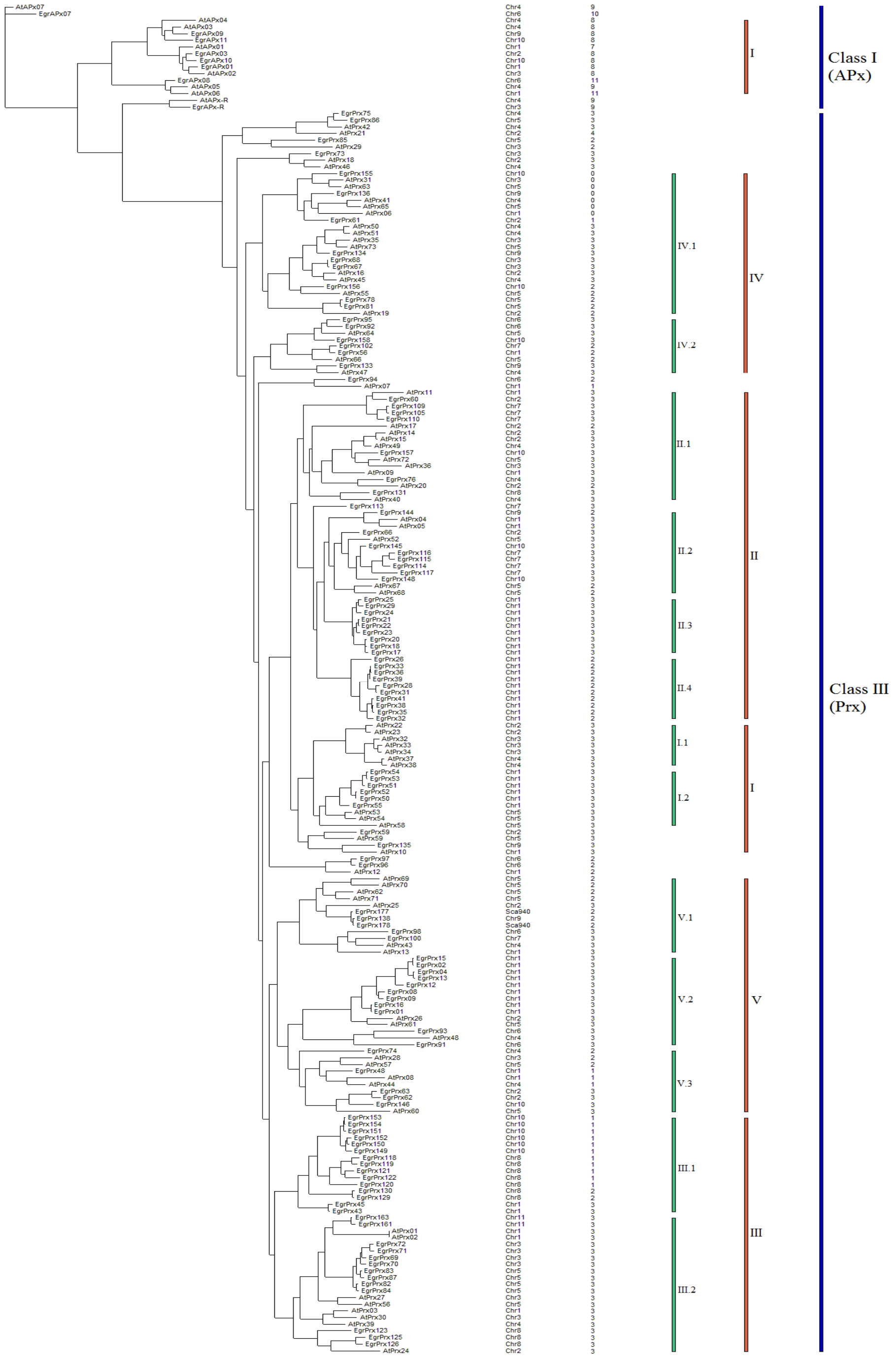

Fig. S11 Heat map of the expression of the APx and CIII Prx genes from *E. grandis* in seven different tissues determined by RNA-seq. The chromosomal localization of each gene was listed on the right.

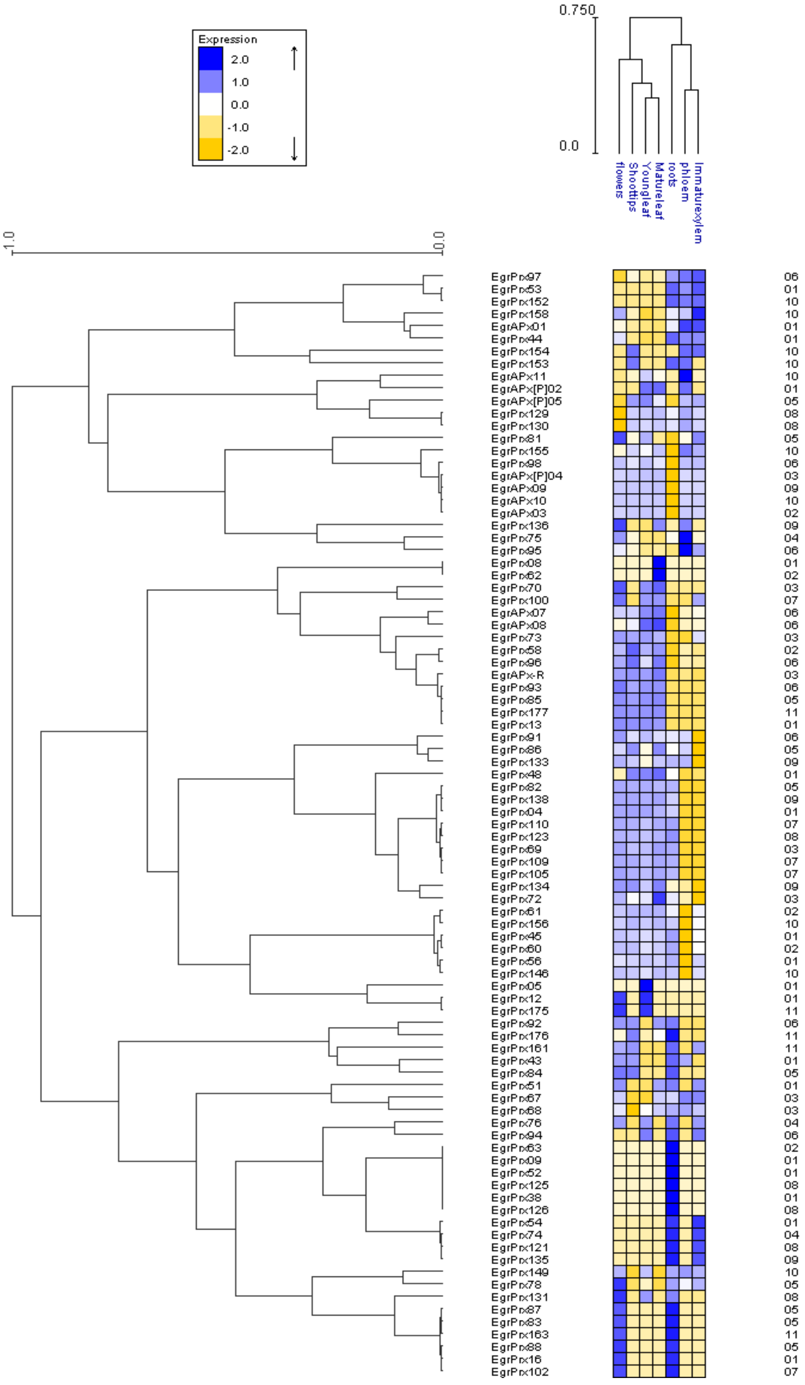

Supplement: Supplementary Data [file supp_evv048_Supplementary_Files_S1_Figures_S1-S11_GBE.pdf]
