# Supplementary material for: Explosive Tandem and Segmental Duplications of Multigenic Families in Eucalyptus grandis
Source: Genome Biol Evol. 2015 Mar 13;7(4):1068–81. doi: 10.1093/gbe/evv048 (PMC4419795; doi:10.1093/gbe/evv048)
Supplement: Supplementary Data [file supp_evv048_New_Microsoft_Office_Word_Document.docx]

**Supplementary_Files_S1_Figures_Legends**

**Fig S1** **Phylogenetic representation of AP2 proteins from *E. grandis* and *A. thaliana*.**

(a) sub-family AP2; (b) sub-family RAV; (c) sub-family DREB; (d) sub-family ERF. The bootstrap values are written at the tree nodes. The clustering of sub-family DREB and ERF were analyzed and marked with vertical lines and cluster (sub-cluster) names in different colors in (c) and (d).

**Fig S2** **Heat map of the expression of the AP2/ERF genes from *E. grandis* in seven different tissues determined by RNA-seq.**

The chromosomal localization of each gene is listed on the right.

**Fig S3** **Phylogenetic representation of auxin transporters PIN (A) and AUX/LAX (B) proteins from *E. grandis* and *A. thaliana*.**

The bootstrap values are written at the tree nodes. Intron numbers and the chromosomal localization of each gene are listed on the right of the trees. The gene structures are also visualized on the right sides. The clustering of family PIN was analyzed and marked with vertical lines and cluster (sub-cluster) names in different colors in (a).

**Fig S4** **Phylogenetic representation of DNAj proteins from *E. grandis* and *A. thaliana.***

Schematic radial phylogeny (a) and horizontal cladogram and (b) of the whole set of DNAj. Bootstrap values are written at the tree nodes.

**Fig S5** **Heat map of the expression of the DNAj genes from *E. grandis* in seven different tissues determined by RNA-seq.**

The chromosomal localizations of each gene are listed on the right.

**Fig S6** **Phylogenetic representation of GRAS proteins from *E. grandis* and *A. thaliana.***

Bootstrap values are written at the tree nodes.

**Fig S7** **Heat map of the expression of the GRAS genes from *E. grandis* in seven different tissues determined by RNA-seq.**

The chromosomal localization of each gene is listed on the right.

**Fig S8** **Phylogenetic representation of LEA proteins from *E. grandis* and *A. thaliana.***

A schematic radial phylogeny of the whole set of LEAs (a) and its horizontal cladogram (b). The LEA2 family is represented in dark blue, LEA5 in azure, LEA6 in violet, LEA/DNH in orange, LEA/SMP in pink, LEA1 in green, LEA3 in red and the common root in black. Bootstrap values are written at the tree nodes (b).

**Fig S9** **Heat map of the expression of the LEA genes from *E. grandis* in seven different tissues determined by RNA-seq.**

The chromosomal localization of each gene is listed on the right.

**Fig S10** **Phylogenetic representation of peroxidase family: APx and CIII Prx proteins from *E. grandis* and *A. thaliana.***

The chromosome localization and introns number of each gene are written on the right of the tree. The clustering of APx and CIII Prx families were analyzed and marked with vertical lines and cluster (sub-cluster) names in different colors.

**Fig S11** **Heat map of the expression of the APx and CIII Prx genes from *E. grandis* in seven different tissues determined by RNA-seq.**

The chromosomal localization of each gene is listed on the right.

**Supplementary_Files_S2_Tables_Legends**

**Table S1 List of AP2 genes in *E. grandis.***

The new proposed nomenclature, sub-class memberships, CBF name, old and new Phytozome nomenclatures, scaffold localization (start and stop codons), scaffold, introns number, quality of the annotation and ESTs number from NCBI, mapped RNAseq (SRR1230929, SRR1015902) and calculated FPKM are displayed. Ok: the sequence is correctly annotated; Bad prediction: the sequence is badly predicted; Partial 3’/5’: Only 3’/5’ end has been annotated; No annotation: no annotation has been found from Phytozome; nd: not detected; Removed: the record has been removed from Phytozome.

**Table S2 List of auxin transporters: PIN and AUX/LAX genes in *E. grandis.***

The new proposed nomenclature, sub-class memberships, old and new Phytozome nomenclatures, scaffold localization (start and stop codons), scaffold and introns number, quality of the annotation and ESTs number from NCBI, mapped RNAseq (SRR1230929, SRR1015902) and calculated FPKM are displayed. Ok: the sequence is correctly annotated; Bad prediction: the sequence is badly predicted; Partial 3’/5’: Only 3’/5’ end has been annotated; No annotation: no annotation has been found from Phytozome; nd: not detected.

**Table S3 List of DNAj genes in *E. grandis.***

The new proposed nomenclature, sub-class memberships, old and new Phytozome nomenclatures, scaffold localization (start and stop codons), scaffold and introns number, quality of the annotation and ESTs number from NCBI, mapped RNAseq (SRR1230929, SRR1015902) and calculated FPKM are displayed. Ok: the sequence is correctly annotated; Bad prediction: the sequence is badly predicted; Partial 3’/5’: Only 3’/5’ end has been annotated; No annotation: no annotation has been found from Phytozome; nd: not detected.

**Table S4 List of GRAS genes in *E. grandis.***

The new proposed nomenclature, sub-class memberships, old and new Phytozome nomenclatures, scaffold localization (start and stop codons), scaffold and introns number, quality of the annotation, ESTs number from NCBI, mapped RNAseq (SRR1230929, SRR1015902) and calculated FPKM are displayed. Ok: the sequence is correctly annotated; Bad prediction: the sequence is badly predicted; Partial 3’/5’: Only 3’/5’ end has been annotated; No annotation: no annotation has been found from Phytozome; nd: not detected; Removed: the record has been removed from Phytozome.

**Table S5 List of LEA genes in *E. grandis.***

The new proposed nomenclature, sub-class memberships, old and new Phytozome nomenclatures, scaffold localization (start and stop codons), scaffold and introns number, quality of the annotation and ESTs number from NCBI, mapped RNAseq (SRR1230929, SRR1015902) and calculated FPKM are displayed. Ok: the sequence is correctly annotated; Bad prediction: the sequence is badly predicted; Partial 3’/5’: Only 3’/5’ end has been annotated; No annotation: no annotation has been found from Phytozome; nd: not detected; Removed: the record has been removed from Phytozome.

**Table S6 List of APx and CIII Prx genes (Non Animal peroxidase) in *E. grandis.***

The PeroxiBase ID, new proposed nomenclature, sub-class memberships, status, old and new Phytozome nomenclatures, scaffold localization (start and stop codons), scaffold and introns number, quality of the annotation and ESTs number from NCBI, mapped RNAseq (SRR1230929, SRR1015902) and calculated FPKM are displayed. Ok: the sequence is correctly annotated; Bad prediction: the sequence is badly predicted; Partial 3’/5’: Only 3’/5’ end has been annotated; PS: Partial sequence without both 3' and 5' ends; No annotation: no annotation has been found from Phytozome; nd: not detected.

**Table S7 The RNA-seq data of the mutigenic families in *E. grandis*.**

In the RNA-seq the RNA from immature xylem, young leaf, mature leaf, shoot tips, roots, flowers, phloem were sequenced. The DNA sequences of genes in families AP2/ERF, PIN, LAX, DNAj, GRAS, LEA, APx and CIII Prx annotated by Phytozome were used to screen the RNA-seq data. Value of zero stands for genes with no hit (no expression) and empty cells correspond to genes not included in the mapping process. FPKM calculated from publicly available RNAseq (SRR1230929, SRR1015902) is also displayed
